# Co-interaction of nitrofuran antibiotics and the saponin-rich extract on gram-negative bacteria and colon epithelial cells

**DOI:** 10.1007/s11274-023-03669-2

**Published:** 2023-06-05

**Authors:** Adam Grzywaczyk, Wojciech Smułek, Anna Olejnik, Urszula Guzik, Agnieszka Nowak, Ewa Kaczorek

**Affiliations:** 1 Institute of Chemical Technology and Engineering, Poznan University of Technology, Berdychowo 4, 60-695 Poznan, Poland; 2 Department of Biotechnology and Food Microbiology, Poznań University of Life Sciences, Wojska Polskiego, 48, 60-627 Poznań, Poland; 3 Institute of Biology, Biotechnology and Environmental Protection, Faculty of Natural Science, University of Silesia in Katowice, Jagiellonska 28, 40-032 Katowice, Poland

**Keywords:** Antibiotics, Cytotoxicity, Fatty acids, Natural surfactants, Pharmaceutics

## Abstract

Large-scale use of nitrofurans is associated with a number of risks related to a growing resistance to these compounds and the toxic effects following from their increasing presence in wastewater and the environment. The aim of the study was to investigate an impact of natural surfactant, saponins from *Sapindus mukorossi*, on antimicrobial properties of nitrofuran antibiotics. Measurements of bacterial metabolic activity indicated a synergistic bactericidal effect in samples with nitrofurantoin or furazolidone, to which saponins were added. Their addition led to more than 50% greater reduction in viable cells than in the samples without saponins. On the other hand, no toxic effect against human colon epithelial cell was observed. It was found that exposure to antibiotics and surfactants caused the cell membranes to be dominated by branched fatty acids. Moreover, the presence of saponins reduced the hydrophobicity of the cell surface making them almost completely hydrophilic. The results have confirmed a high affinity of saponins to the cells of *Pseudomonas* strains. Their beneficial synergistic effect on the action of antibiotics from the nitrofuran group was also demonstrated. This result opens promising prospects for the use of saponins from *S. mukorossi* as an adjuvant to reduce the emission of antibiotics into the environment.

**Supplementary Information:**

The online version contains supplementary material available at 10.1007/s11274-023-03669-2.

## Introduction

Nitrofurantoin (NFT), nitrofurazone (NFZ), furaltadone (FTD) and furazolidone (FZD), i.e. antibiotics from the nitrofuran group, are recognized as a group of the most popular antimicrobials all over the world. The nitrofurantoin only has been prescribed 2,957,359 times in 2019 in the USA, which is not an impressive result, but it can still be concluded that nitrofuran drugs are widely used (Nitrofurantoin Drug Usage Statistics, United States, 2013–2020, 2020). NFT has been used in the treatment of the urinary tract, while FZD has been used to treat diarrhea, cholera and bacteremic salmonellosis. In 1995, their use as a food additive for farm animals was banned due to concerns about the carcinogenicity of the drug residues and their potentially harmful effects on human health (Vass et al. 2008).

The increase in antibiotic intake is one of the main reasons for the increase in antimicrobial resistance (AMR). Increasing therapeutic doses, forced by greater drug resistance of pathogenic strains, contributes to the increasing presence of antibiotics in the environment. This phenomenon is a feedback loop, as it results in the spread of antibiotic resistance (Polianciuc et al. 2020). This process can be interrupted by increasing the bioavailability of the antibiotic, allowing the use of lower doses of the antibiotic while maintaining the expected effectiveness (Price and Patel 2022). Moreover, the World Health Organization (WHO) has published a list of bacteria that are characterized by high drug resistance so that effective treatments for infections caused by them are running out (Asokan et al. 2019). At the top of the list are bacteria of the genus *Acinetobacter baumannii, Enterobacteriaceae*, and also *Pseudomonas aeruginosa* (Ropponen et al. 2021).

One of the possible methods of increasing drug bioavailability is to modify the permeability of the cell membrane with surface active properties (Smułek and Kaczorek 2022). One group of surfactants that is attracting increasing attention are saponins, natural surfactants of natural origin (Liao et al. 2021). The structure of their molecules is most likely responsible for the ability to integrate the hydrophobic part of their molecules into the structure of the cell membrane, while the hydrophilic part remains on the surface. There are also suggestions that saponin monomers can incorporate into the outer part of the membrane and increase the distance between membrane components on the surface, which leads to a positive membrane curvature and the formation of specific domains, whose size increases with time (Rojewska et al. 2023). The presence of sugar chains helps develop membrane defects and gradually increases the membrane permeability, even by creating holes in the membrane (Lorent et al. 2014; Rojewska et al. 2020).

The aim of our study was to determine the influence of the co-action of saponin-rich *Sapindus mukorossi* extract and nitrofuran antibiotics: nitrofurantoin (NFT) and furazolidone (FZD), on the cellular response of gram-negative bacteria of the *Pseudomonas* genus. *Pseudomonas aeruginosa* is outside the action spectrum of nitrofurans, and therefore, we were able to observe the properties of living cells and the effect of drug-saponin interaction on the cell membrane. The toxicity of the saponin extract on human intestinal epithelial cells was also assessed. Intestinal cells are the first barrier limiting drug penetration into the body from gastrointestinal tract. Analysis of the influence of saponins on these cells may help establish ways of reusing nitrofuran antibiotics in combination with biosurfactants. As absorption of nitrofurans by the human body is severely restricted, it is important to check saponins’ toxic effects on the cells constituting the intestinal barrier in the drug absorption process (Huttner and Harbarth 2017). Although there are many studies on the modification of the permeability of model membranes as well as the bactericidal properties of the saponins themselves, there have been few reports so far on the possible synergistic effect between antibiotics and saponins (Jurek et al. 2019; Lorent et al. 2014; Rojewska et al. 2020; Sreij et al. 2018). Such an effect may contribute to achieving a desired therapeutic effect at a lower dose of the drug. Thus, in our study we focused on modifying the properties of the outer membrane of gram-negative bacteria by employing the co-interaction of the antibiotic with saponins. Using Electrophoretic light scattering and Dynamic light scattering, and analysis of Congo Red adsorption and Crystal Violet permeability, we tried to more deeply understand the possible mechanism of interaction of antibiotics and surfactants on the biological membranes. For this purpose, we also performed lipidomic analysis. The performed basic analyses of the metabolic activity of both bacterial cells and human intestinal epithelial cells provide important information about the toxicity and safety of saponins derived from *Sapindus mukkorosi,* and also indicate a possible synergistic effect between chosen antibiotics and saponins. The obtained results provide new, important information on the possible interaction of surfactants with nitrofuran antibiotics and allow a better understanding of their interference in the lipid profile of biological membranes. The use of saponins may contribute to reduction of the growing bacterial resistance to antibiotics through the use of compounds of natural origin.

## Materials and methods

### Chemicals

All chemicals used in the study, including two nitrofuran—NFT and FZD, were of the highest purity grade and were purchased from Merck KGaA (Darmstadt, Germany). The nutrient agar, nutrient broth and other microbiological supplements came from BTL sp. z o.o. (Łódź, Poland). *Sapindus mukorossi* nuts were obtained from Mohani (Psary, Poland). The saponins-rich extract was obtained via methanol extraction as described by Smułek et al. (2016).

### Cytotoxicity analysis

The cytotoxicity of *S. mukorossi* extract combined with antibiotics was assessed using human CCD 841CoN (ATCC^®^CRL-179^™^) cells derived from normal colon mucosa and obtained from the American Type Culture Collection (ATCC, Manassas, VA, USA). The cells were cultured in Dulbecco’s Modified Eagle’s Medium (Sigma-Aldrich, Poznań, Poland) supplemented with 10% fetal bovine serum (Gibco BRL, USA) and 1% nonessential amino acid solution 100 × (Sigma-Aldrich). They were grown at 37 °C in a humidified atmosphere (5% CO_2_, 95% air) and subcultured twice a week after reaching ca. 80% confluence. A trypsin-EDTA solution (0.25%) was used to harvest the CCD 841CoN cells.

In the cytotoxicity experiments, the cells were grown in 96-well plates at the initial density of 1.5 × 10^4^ cells cm^−2^. Twenty-four hour cell cultures were treated with *S. mukorossi* extract at concentrations ranging from 0 to 1000 μg mL^−1^ with the addition of antibiotics at a final concentration of 5 μg mL^−1^. After 48 h of treatment, cell viability and metabolic activity were assessed using the MTT (3-(4,5-dimethylthiazol-2-yl)-2,5-diphenyltetrazolium bromide) test (Sigma-Aldrich), as described by Smulek et al. (2020). Briefly, the MTT solution was added to each well to obtain a concentration of 0.5 mg MTT mL^−1^. The microplate was incubated at 37 °C for 3 h, and then formazan crystals were extracted with acidic isopropanol for 20 min at room temperature. Absorbance was measured at 570 and 690 nm using a Tecan M200 Infinite microplate reader (Tecan Group Ltd., Männedorf, Switzerland).

### Bacteria strain and culture conditions

Three strains of bacteria of the genus *Pseudomonas* were used in the study: *Pseudomonas plecoglossicida* IsA (NCBI GenBank Accession No. KY561350), *Pseudomonas* sp. MChB (NCBI GenBank Accession No. KU563540), *Pseudomonas* sp. OS4 (NCBI GenBank Accession No. KP096512). The bacteria were stored on nutrient agar plates. For incubation, the bacterial biomass was suspended in a nutrient broth until it reached the exponential growth phase. The bacterial cultures were then centrifuged at 4500 rcf and re-suspended in a PBS (**P**hosphate-**B**uffered **S**aline) solution at a constant neutral pH.

A growth curves of pure bacteria cultures and in presence of xenobiotics are presented in Supplementary Data with additional explanation. The measurements were performed with a microplate reader (Multiskan 152 Sky, Thermo Fisher Scientific, Waltham, MA, USA) and the 96-well clear bottom sterile microplates as described by Pacholak et al. (2023) 100 μL of the prepared bacterial cultures were transferred to the microplate wells. The plates were maintained at 30 °C with pulse shaking. The OD_600_ of each well were read every 10 min for 24 h.

In order to determine the action of 5 mg L^−1^ FZD and NFT antibiotics in combination with 10 mg L^−1^ of saponins or without saponins on bacterial cells, liquid cultures were prepared. The bacteria were centrifuged from growth medium and then re-suspended in the 1 mL of described mixtures for 24 h. The control sample consisted of the bacteria suspended in the PBS solution. After 24 h, the tests described in the further experiments were carried out.

### Fatty acids profile of bacterial strains

The procedure of fatty acid methyl ester extraction, analysis and identification using gas chromatography, and data interpretation were analogous to the methodology described by Nowak and Mrozik (2016). The mean fatty acid chain length was expressed by the following equation:

$${\text{Mean}}\, {\text{fatty}}\, {\text{acid chain}}\, {\text{lenght}} = \sum \left( {\% FA \times C} \right)/100$$

where: %FA is the percentage of fatty acid, and C is the number of carbon atoms. To prevent the alterations caused by fatty acids occasionally detected, the analysis of FAMEs included only fatty acids with a content of at least 1%.

The obtained results were evaluated by analysis of variance, and statistical analyses were performed on three biological replicates of data obtained from each treatment. The statistical significance (*p* < 0.05) of differences was treated by one-way ANOVA, considering: (1) the effect of each treatment on tested bacterial strains and (2) the influence of NFT and FZD on each bacterial strain. Next, differences between particular samples were assessed by post-hoc comparison of means using the lowest significant difference (LSD) test.

### Cell surface properties measurements

The above analyses were performed on cultures of bacteria suspended for 24 h in solutions containing the drug and saponins. The analysis of cell surface properties included evaluation of Congo red binding to microbial cells, according to Ambalam et al. (2012). Moreover, the cell membrane permeability test using crystal violet was performed, as well as an MTT enzymatic activity test, as described by Smulek et al. (2020). The zeta potential was calculated from the Smoluchowski equation after measurements of electrophoresis mobility using a Zetasizer Nano ZS instrument (Malvern Instruments Ltd. UK). Additionally, the cells’ sizes were measured using a Mastersizer 2000 instrument (Malvern Instruments Ltd.) equipped with a Hydro 2000S unit that enables the analysis of samples in the form of a wet dispersion. The cells diameters were measured in the range of 0.02–2000 μm. For this purpose, an appropriate quantity of the material was dispersed in a water medium, and after establishing the instrument background, appropriate measurements were made. An atomic force microscope Park NX10 from Park Systems (Suwon, South Korea) was used to analyze changes in the cell topography of the bacteria as described by Pacholak et al. (2023).

### Statistical analysis

The results presented in the study were calculated as an average value from at least three independent experiments. The variance analysis and t-Student test were applied to determine the statistical significance of differences between the average values. The differences were considered statistically significant at *p* < *0.05*. All calculations were conducted using Excel 2019 (Microsoft Office) software. The FAME profiles were also subjected to principal component analysis (PCA). All analyses were performed using the Statistica 13.3 PL software package.

## Results and discussion

### Cytotoxicity analysis

**Fig. 1:**
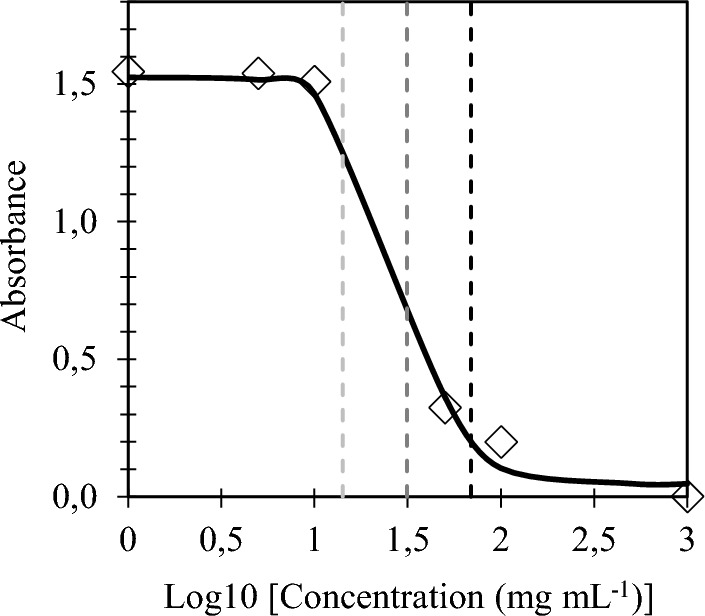
Dose–response curve for normal colon CCD 841CoN cells treated with S. *mukorossi* extract at concentrations ranging from 1 to 1000 g mL^−1^. Cell viability was measured by MTT assay. IC_10_; IC_50_; IC_90_

Cytotoxicity of saponins from S. *mukorossi* and nitrofuran antibiotics was determined in normal human cells to evaluate their safety in antibiotic therapy. The effect of saponins-rich plant extract on the proliferation, viability, and metabolic activity of colon epithelial CCD 841CoN cells is shown in Fig. 1.

**Fig. 2:**
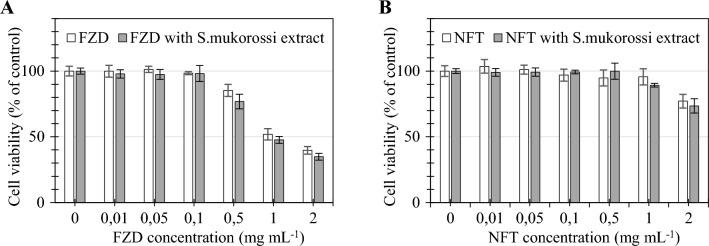
Effect of FZD **A** and NFT **B** with and without *Sapindus mukorossi* extract at a concentration of 10 µg mL^−1^ on the viability of human colon mucosa CCD 841CoN cells

The experiments revealed that *S. mukorossi* saponin extract at concentrations up to 10 μg mL^−1^ is not cytotoxic to the normal intestinal cells. The lowest cytotoxic dose, that is the concentration of the extract causing a decrease in cell viability by 10%, was calculated as 16.79 ± 2.87 µg mL^−1^ (Table 1). Figure 1 shows a dose–response relationship between saponin-rich extract and colon mucosa cells. Based on the experimental results and model fitting, a half maximal inhibitory concentration (IC_50_) was determined at 32.72 ± 2.63 µg mL^−1^. For comparison, the IC_50_ of the antibiotics were estimated to be 1.28 µg mL^−1^ and 2.92 µg mL^−1^ for FZD and NFT, respectively (Table 1). Notably, the saponin extract combined at the concentration of 10 µg mL^−1^ with the antibiotics did not significantly affect their cytotoxicity; the extract supplementation did not change the cytotoxic potential of the antibiotics at every concentration tested (Fig. 2). The obtained results indicate that the use of *S. mukorossi* saponins at non-cytotoxic doses does not cause any additional cytotoxic effect on normal human colon cells. Therefore, the concentration of saponin extract of 10 µg mL^−1^ was chosen for further experiments.

**Table 1:** Cytotoxic doses expressed as inhibitory concentrations (IC) of *Sapindus mukorossi* extract and/or nitrofuran antibiotics determined in human normal colon mucosal CCD 841CoN cells. Data present first (IC_10_), half-maximal (IC_50_) and lethal (IC_90_) inhibitory concentrations

Extract and/or antibiotic	IC (μg mL^−1^)		
	IC_10_	IC_50_	IC_90_
*S. mukorossi* extract	16.79 ± 2.87	32.72 ± 2.63	64.12 ± 1.57
FZD	0.33 ± 0.06^a^	1.28 ± 0.11^b^	4.98 ± 0.29^d^
FZD *S. mukorossi* 10 μg mL^−1^	0.27 ± 0.02^a^	1.13 ± 0.07^b^	4.22 ± 0.30^d^
NFT	1.43 ± 0.12^b^	2.92 ± 0.02^c^	6.02 ± 0.51^e^
NFT *S. mukorossi* 10 μg mL^−1^	1.28 ± 0.12^b^	2.94 ± 0.16^c^	6.76 ± 0.17^e^

Values marked with the same letter do not differ significantly (*p* > 0.05)

As follows from peruse of literature, the results presented in our study are one of the few concerning the impact of saponin-rich extracts on unmutated human cells. The vast majority of studies reported to date have described the cytotoxicity of saponins mainly against cancer cells to assess their antitumor potential. For example, Hashemi et al. (2021) have observed that ginsenosides belonging to the saponin group have the ability to induce apoptosis and arrest the cell cycle. Moreover, gypensapogenin H derived from *Gynostemma pentaphyllum* significantly inhibited the growth of human breast cancer cells (MDA-MB-231), while exhibiting low toxicity to normal human breast epithelial MCF-10a cells (Zhang et al. 2015). Zhang et al. (2022) have reported that asiaticoside saponin at concentrations of 20, 40, and 80 µg mL^−1^ is not toxic to human retinal pigment epithelium ARPE-19 cells. Duewelhenke et al. (2007) have indicated the suppression of the proliferation of primary human osteoblasts by antibiotics fluoroquinolones, macrolides, clindamycin, chloramphenicol, rifampin, tetracycline, and linezolid at doses up to 20 or 40 µg mL^−1^. It has been suggested that a significant problem of the group of antibiotics, such as fluoroquinolones, is that they cause mitochondrial dysfunction induced by oxidative stress in human cells (Nadanaciva et al. 2010; Xiao et al. 2019).

### Bacterial cells viability

The strains utilized in this study exhibit high resistance to both antibiotics. The Minimal Inhibitory Concentration (MIC) for NFT and FZD against the IsA strain reached 100 µg mL^−1^, while for the OS4 and MChB strains, it exceeds 200 µg mL^−1^. Given the solubility limitations of these antibiotics in an aqueous environment, achieving higher concentrations is not feasible. Our investigation involved the addition of *Sapindus mukorossi* extract at concentrations up to 30 µg mL^−1^, which demonstrated no impact on bacterial growth and division (see Supplementary Data). This confirms the absence of a cytostatic effect of the extract on the selected *Pseudomonas* strains. However, in the presence of *Sapindus mukorossi* extract MIC for NFT decreased the MIC nearly 20% for all tested strains, however, for FZD no significant decrease was observed.

**Fig. 3:**
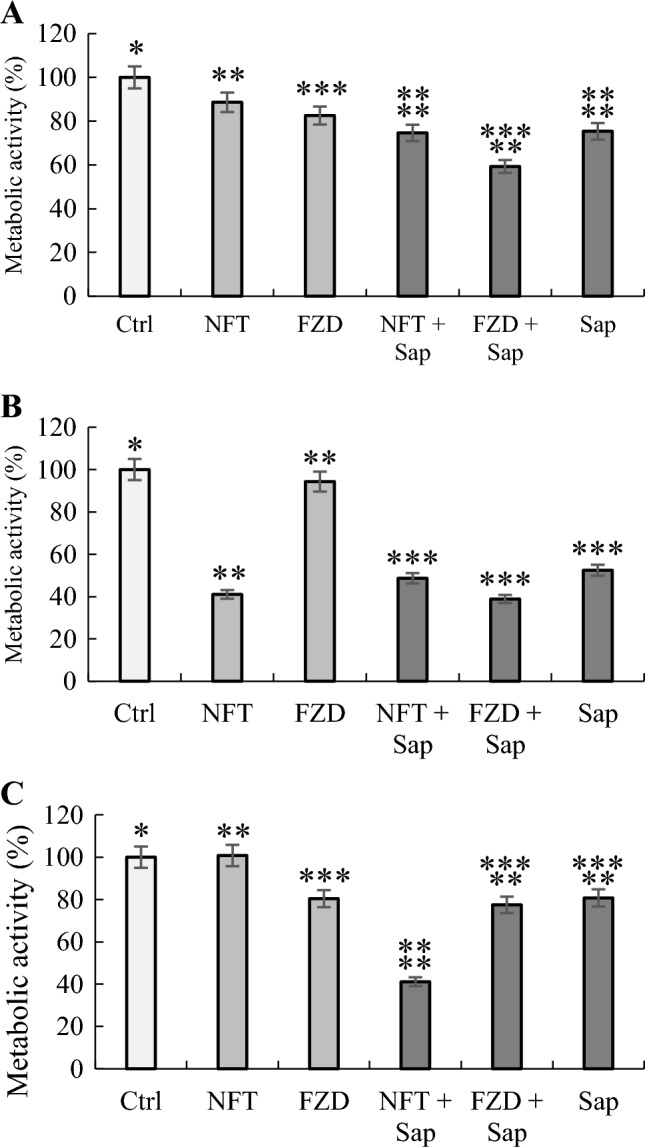
Relative cell metabolic activity of *Pseudomonas* strains in response to different antibiotics and/or *S. mukorossi* extracts; A—*Pseudomonas* sp. OS4, B—*Pseudomonas plecoglossicida* IsA, C—*Pseudomonas* sp. MChB, Ctrl—control culture (without antibiotics and plant saponins), NFT—nitrofurantoin at 5 mg L^−1^, FZD—furazolidone at 5 mg L^−1^, Sap – *Sapindus mukorossi* plant saponins at 10 mg L^−1^

To investigate deeper, the saponins-antibiotics co-action, the enzymatic activity of *Pseudomonas* spp. was assessed by MTT assay (Smułek et al. 2020) and the results are presented in Fig. 3. For each tested strain, a decrease in the enzymatic activity was observed after the FZD antibiotic addition. Interestingly, a very slight increase in the activity of *Pseudomonas* sp. MChB strain exposed to NFT was observed, which may suggest no toxic effect in this case. However, the addition of saponins caused a significant decrease in the enzymatic activity relative to that of the samples without the saponins added. The highest decrease in enzymatic activity was observed for *P. plecoglossicida* IsA strain, of even up to 39% relative to that of the control sample. The addition of saponins to the NFT also caused a toxic effect (a decrease in activity of cells by 41% relative to that of the control sample) on the *Pseudomonas* sp. MChB strain, on which the drug itself did not cause such an effect. Such results may suggest the presence of a synergistic effect between nitrofuran antibiotics and saponins, which enhance the toxic effect of the antibiotic. Moreover, pure saponins also caused a decrease in the metabolic activity of *P. plecoglossicida* IsA strain of up to 52% compared to that of the control. Both phenomena may be related to the formation of holes and the incorporation of saponins into the outer cell membrane, which is the first barrier between the bacterial cell and the environment. Zaynab et al. (2021) have suggested that saponins have a toxic effect on both gram-positive and gram-negative bacteria as well as fungi. The results obtained in our study correlate well with those obtained by Khan et al. (2018). The saponins obtained from green tea seeds show antibacterial activity against *Escherichia coli*, *Salmonella* spp., and *Staphylococcus aureus*. For most of the tested strains, a decrease in OD_600_ to a value close to 0 was observed for the saponin concentration equal to the minimal inhibitory concentration, as well as a decrease in OD_600_ with increasing concentration of saponins. On the other hand, Zdarta et al. (2019) have observed no toxic effect of saponins derived from *Hedera helix*, and even their stimulating effect towards *Raoultella ornithinolytica* and *Achromobacter calcoaceticus*. A fairly large variety of saponins, as well as the presence of a sugar part in the molecule, which can be used as an alternative carbon source for bacteria, determine the possibility of obtaining different results of toxicity assessment for different bacteria strains.

Regarding cell morphology (refer to AFM images in Supplementary Data) of the control samples, a regular structure without defects and uniformity in all directions can be observed. The dimensions of the cells for all strains are approximately 2 µm in length. Notably, exposure to NFT resulted in distinct changes such as visible furrows and irregularities, particularly prominent in the IsA and OS4 strains, and to a lesser extent in MChB. The addition of saponins does not appear to have a significant impact on cell topography, as the effects are primarily attributed to NFT itself. These changes may indicate cellular damage caused by the antibiotic; however, complete cell lysis is not evident, as supported by the obtained growth curves that indicate no cytostatic effect. Similar observations were made by Pacholak et al. (2023), who found that *Stenotrophomonas acidaminiphila* N0B, *Pseudomonas indoloxydans* WB, and *Serratia marcescens* ODW152 exhibit discernible alterations in cell structure after prolonged exposure to nitrofurantoin, with greater severity observed after 28 days.

### Bacterial fatty acid profile

**Fig. 4:**
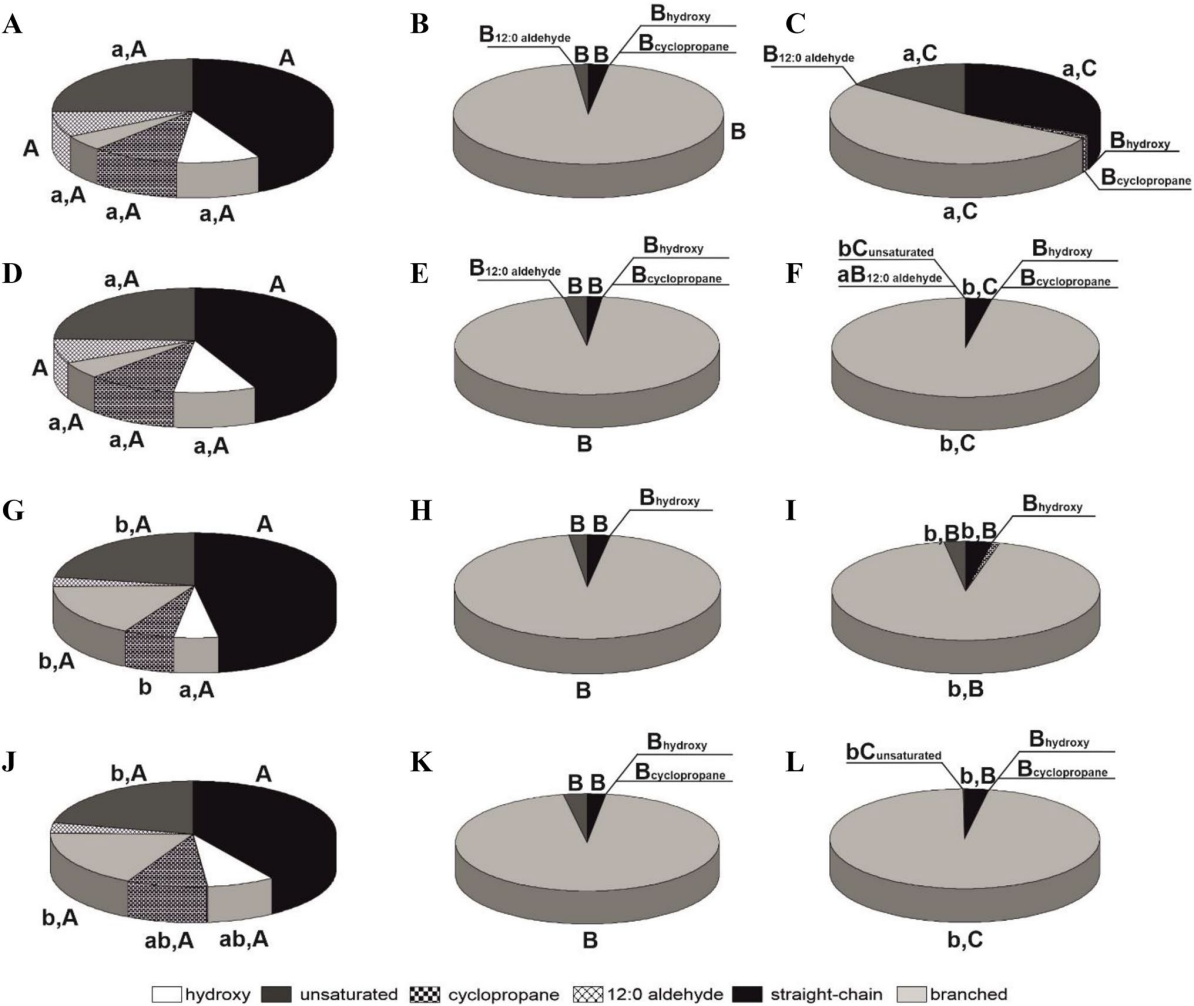
Proportions of fatty acids in IsA (**A**, **D**, **G**, **J**), MChB (**B**, **E**, **H**, **K**), and OS4 (**C**, **F**, **I**, **L**) growing on nutrient broth (**A**, **B**, **C**), and nutrient broth supplemented with Sap (**D**, **E**, **F**), nutrient broth supplemented with Sap and FZD (**G**, **H**, **I**), and nutrient broth supplemented with Sap and NFT (**J**, **K**, **L**). The class of hydroxyl fatty acids contains additionally the branched hydroxyl fatty acids. In each column, samples with the same bacterial strain but with different treatments (with or without Sap, NFT and FZD) with different lowercase are significantly different (*p* < *0.05*, LSD test). It means that lowercase indicates differences in FAME profiles within the same bacterial strain. Results without any lowercase are statistically equal at a significance level *p* < *0.05*. The means with the same treatment with a different capital letter are significantly different (*p* < *0.05*, LSD test) considering the effect of treatment (addition of NFT, FZD, or without additional carbon source) between tested bacterial strains. It means that one test was done for control samples (**A**, **B**, **C**), the second for samples with Sap (**D**, **E**, **F**), the third for samples with Sap and furazolidon (**G**, **H**, **I**)), and fourth for samples with Sap and NFT (**J**, **K**, **L**)

**Fig. 5:**
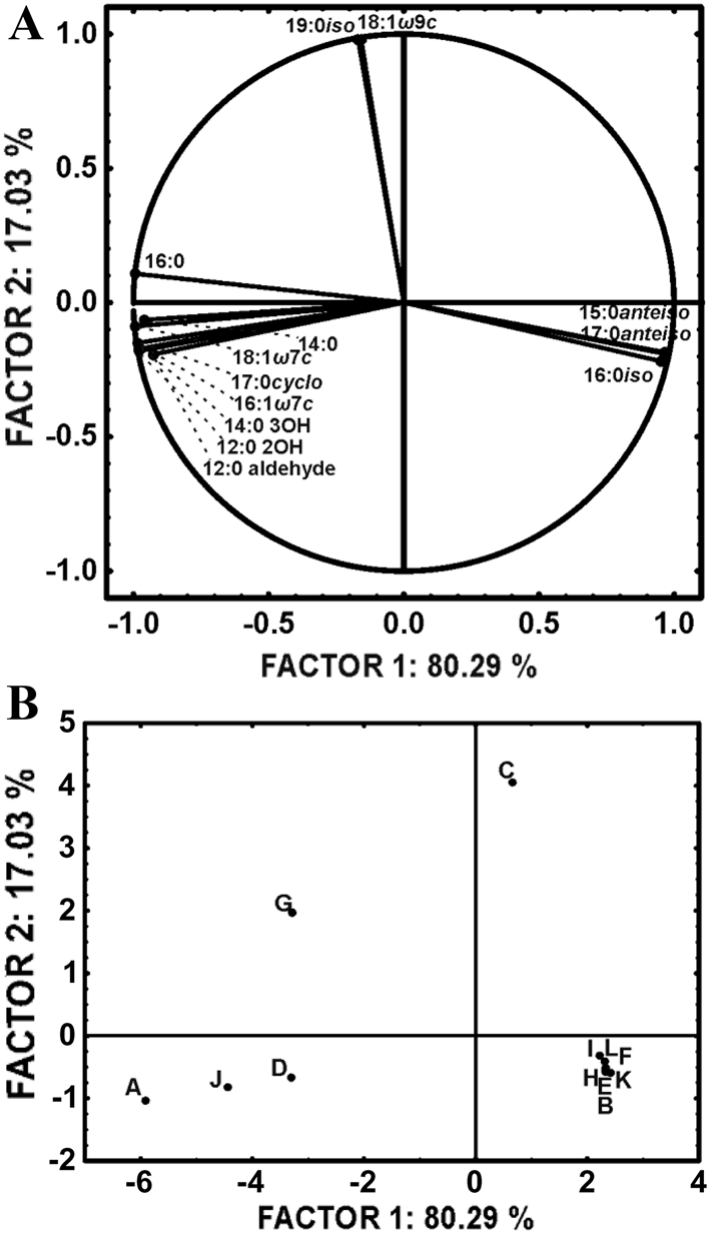
PCA analysis of fatty acids profile of bacterial strains in response to different antibiotics and/or *S. mukorossi* extracts. The test systems are marked with capital letters: A – IsA strain growing on nutrient broth; B—MChB strain growing on nutrient broth; C – OS4 strain growing on nutrient broth; D—IsA strain growing on nutrient broth supplemented with Sap; E—MChB strain growing on nutrient broth supplemented with Sap; F – OS4 strain growing on nutrient broth supplemented with Sap; G—IsA strain growing on nutrient broth supplemented with Sap and FZD; H—MChB strain growing on nutrient broth supplemented with Sap and FZD; I – OS4 strain growing on nutrient broth supplemented with Sap and FZD; J—IsA strain growing on nutrient broth supplemented with Sap and NFT; K—MChB strain growing on nutrient broth supplemented with Sap and NFT; L—OS4 strain growing on nutrient broth supplemented with Sap and NFT

Because the study focuses on bacterial cells membrane and wall properties, the first step of the study was to determine the profile of membrane fatty acids (FA) and the reaction of the strains to the contact with antibiotics. Figure 4 and Table 2 present the share of particular groups of FA in the membranes. In *Pseudomonas plecoglossicida* IsA strain, the straight-chain and saturated FA dominated. The branched FA had the relatively smallest share in the total amount of FA. In the case of two other strains, *Pseudomonas* sp. MChB *Pseudomonas* sp. OS4, the branched FA accounted for over 90% and 50% of total FA, respectively. In contrast to IsA and OS4 in whose cell membranes hydroxy and cyclopropane FA can be found, the FA profile of *P.* MChB cell membrane is limited only to unsaturated and 12:0 aldehyde (Fig. 5).

**Table 2:** Mean fatty acid chain length and ratio of saturated to unsaturated fatty acids of tested bacterial strains in response to different antibiotics and/or *S. mukorossi* extracts: a) *Pseudomonas plecoglossicida* IsA, b) *Pseudomonas *sp. OS4, c) *Pseudomonas *sp. MChB

Treatment	Ctrl	Sap	Sap/NFT	Sap/FZD
Strain	*Pseudomonas plecoglossicida* IsA			
Mean fatty acid chain length	16.92 ± 0.06	17.40 ± 0.78	16.14 ± 0.52	15.93 ± 0.61^a^
*Sat./Unsat. ratio*	2.96 ± 0.17	3.08 ± 10.89	3.63 ± 0.07^a^	3.43 ± 0.07^a,b^
Strain	*Pseudomonas* sp. MChB			
Treatment	Ctrl	Sap	Sap/NFT	Sap/FZD
Mean fatty acid chain length	15.72 ± 0.01^a^	15.70 ± 0.01^a,b^	15.68 ± 0.00^c^	15.75 ± 0.02^a,d^
*Sat./Unsat. ratio*	55.14 ± 6.54	34.55 ± 0.56^a^	32.98 ± 0.91	49.54 ± 26.40^a^
Strain	*Pseudomonas* sp. OS4			
Treatment	Ctrl	Sap	Sap/NFT	Sap/FZD
Mean fatty acid chain length	15.67 ± 0.53	15.74 ± 0.00	15.73 ± 0.01	15.95 ± 0.028^a^
*Sat./Unsat. ratio*	5.97 ± 2.08	0.00 ± 0.00^a^	0.00 ± 0.00^a^	43.64 ± 24.11^b^

In each table row, the means with different lowercases are significantly different (*p* < *0.05*, LSD test) considering the effect of treatment (Sap, Sap/NFT, Sap/FZD) on bacterial strain. In each column, the means with the same treatment marked with different letters are significantly different (*p* < *0.05*, LSD test) considering the reactions of bacterial strains. It means that one test was done for Control samples, the second for Sap, third for NFT samples, and the fouth for FZD samples

It should be emphasized that the addition of saponins significantly changed the membrane fatty acid profile for IsA and OS4 strains, but had rather limited influence on MChB strain membrane. After the addition of saponin, the percentage of branched fatty acids in OS4 strain membrane increased from 56 to 94% for Sap/FZD and to 97% for Sap and Sap/NFT, whereas the share of unbranched fatty acids decreased from 35 to 3.06% and 6.75% for Sap/NFT and Sap/FZD, respectively. For MChB strain cell membrane a small fluctuation can be noticed for unsaturated acids after treatment with saponins—the share of unsaturated acids increased from 0.79 to 2.29% for Sap/FZD and 2.9% for Sap and Sap/NFT, at the expanse of branched and straight chain fatty acids. Nonetheless, the share of unsaturated FA was about 95% for each sample of MChB strain. The IsA strain cell membrane has the most complex composition with 45% straight-chain, 10.5% hydroxylated, 11% cyclopropane, 5.5% branched, 8.5% 12:0 aldehyde, and 27.5% unsaturated FAs. In this case, the addition of pure saponin did not change the membrane FA profile. Interestingly, the synergistic effect between saponin and FZD/NFT might occur, and as a result, only in this combination, the branched FA share increased from 5.5% to 18.30% and 16.74% for Sap/NFT and Sap/FZD, respectively. What should also be noted, after Sap/FZD treatment the share of straight chain FA increased to 48%. This also confirmed that bacteria modify their cells biomembrane in response to a toxic environment.

The modulation of the length, branching, and saturation of the FA acyl chains is one of the main bacteria mechanisms in response to stress conditions. The increased amount of unsaturated and branched FA increase membrane fluidity and enhance the diffusion processes through it (Willdigg and Helmann 2021). Such a change can be noticed for IsA and OS4 strains after treatment with saponins. Górny et al. (2019) have studied the interaction of naproxen with *Bacillus thuringiensis* B1 and observed, after contact with the pharmaceutic, a decrease in the content of unsaturated FA, which were replaced by hydroxy FA. Similarly, Pacholak et al. (2021) have found for *Pseudomonas hibiscicola* strain FZD2 that after its contact with NFT and furazolidone, the proportion of branched FA increased at the expense of unsaturated and straight-chain FA. Hence, the results obtained in our study indicate that the tested *Pseudomonas* strains appeared to be relatively resistant to the antibiotics studied, from among which particularly strongly resistant to NFT. This antibiotic seemed to have no significant effect on the cell membrane. What is more, no changes in FA profile after exposed to saponins were observed. Moreover, in *P. plecoglossicida* IsA cell membranes the share of straight-chain FA increased in response to the toxic environment.

### Cell membrane permeability

Low permeability of the bacteria cell membrane is one of the key factors limiting the effectiveness of antibiotics. Lowering the membrane permeability is also one of the possible mechanisms of cellular response to stressful conditions. The cell membrane permeability was assessed using the absorption of crystal violet by bacterial cells, on a scale where 100% meant complete dye absorption, 0% meant no crystal violet absorption and thus, a complete stop of the transport process through the cell membrane. The effect of reducing the permeability of the cell membrane can be observed when treated with pure antibiotic solutions without the addition of saponins (Table 3). The greatest decrease in the dye absorption was obtained for the *Pseudomonas* sp. OS4 strain—from 59.9 to 36.2% for FZD. A subtle increase in cell membrane permeability was obtained for the *P. pleocglossicida* IsA strain from 65.6 to 66.4%. Such a small increase is within the limit of the measurement error and may indicate practically no effect. A significant increase in the permeability for each of the tested strains was observed after adding the extract of *S. mukorossi*. In the case of the *P. plecoglossicida* IsA strain, the obtained results suggested an increase in the permeability of the cell membrane by about 20–25 percentage points as compared to the control sample. Comparing the results for pure antibiotics, the addition of saponins increased the permeability by 23 percentage points for *P. plecoglossicida* IsA exposed to FZD or FZD and *S. mukorossi* extract, and even by 53 percentage points for the *Pseudomonas* sp. MChB strain and the NFT drug. Knudsen et al. (2008) have come to similar conclusions. In their study they observed that the presence of soyabean saponins increase the intestinal epithelial permeability as determined by both reduced transepithelial resistance and increased apparent permeability of [^14^C]mannitol (Knudsen et al. 2008). The increase in permeability may be related, as suggested by Jacob et al. (1991) and Zheng and Gallot (2021), to the ability of saponins to solubilize cholesterol, thereby creating tears without disturbing the remaining structure of the biomembrane. Sudji et al. (2015) on the other hand, have suggested that it is the presence of cholesterol in the cell membrane that determines the action of saponins. The absence of cholesterol in the cell membrane means that saponins do not have the effect of increasing the membrane permeability (Sudji et al. 2015).

**Table 3:** Cell membrane permeability and cell surface properties of tested strains after exposure to antibiotics and/or *S. mukorossi* extract; A – *Pseudomonas* sp. OS4, B – *Pseudomonas plecoglossicida* IsA, C – *Pseudomonas* sp. MChB, Ctrl – control culture (without antibiotics and plant saponins), NFT – nitrofurantoin at 5 mg L^-1^, FZD – furazolidone at 5 mg L^-1^, Sap – *Sapindus mukorossi* plant saponins at 10 mg L^-1^

	*Pseudomonas* sp. OS.4	*Pseudomonas plecoglossicida* IsA	*Pseudomonas* sp. MChB
Cell membrane permeability [%]			
Ctrl	58.9 ± 2.9^a^	65.6 ± 3.3^a^	58.5 ± 2.9^a^
NFT	44.3 ± 2.2^b^	45.4 ± 2.3^b^	34.6 ± 1.7^b^
FZD	36.2 ± 1.8^c^	66.4 ± 3.3^a^	49.6 ± 2.5^c^
NFT + Sap	84.1 ± 4.2^d^	84.1 ± 4.2^c^	86.4 ± 4.3^d^
FZD + Sap	87.8 ± 4.4^d^	89.5 ± 4.5^c^	87.5 ± 4.4^d^
Sap	84.6 ± 4.2^d^	90.3 ± 4.5^c^	87.0 ± 4.4^d^
Cell surface adhesivity [%]			
Ctrl	64.9 ± 3.2^a^	71.4 ± 3.6^a^	71.2 ± 3.6^a^
NFT	51.4 ± 2.6^b^	68.3 ± 3.4^a^	72.0 ± 3.6^a^
FZD	62.3 ± 3.1^a^	69.7 ± 3.4^a^	65.3 ± 3.3^a^
NFT + Sap	< 2.0^c^	34.3 ± 1.7^b^	< 2.0^b^
FZD + Sap	< 2.0^c^	15.0 ± 0.7^c^	6.0 ± 0.3^c^
Sap	< 2.0^c^	24.8 ± 1.2^d^	29.9 ± 1.5^d^
Zeta potential [mV]			
Ctrl	− 10.1 ± 0.5^a^	− 12.3 ± 0.6^a^	− 14.5 ± 0.7^a^
NFT	− 9.8 ± 0.5^a^	− 9.7 ± 0.5^b^	− 13.6 ± 0.7^a^
FZD	− 9.5 ± 0.5^a^	− 8.8 ± 0.4^b^	− 14.8 ± 0.7^a^
NFT + Sap	− 36.1 ± 1.8^b^	− 27.2 ± 1.4^c^	− 32.3 ± 1.6^b^
FZD + Sap	− 33.8 ± 1.7^b^	− 24.9 ± 1.2^c^	− 32.6 ± 1.6^b^
Sap	− 17.7 ± 0.9^c^	− 16.2 ± 0.8^d^	− 17.6 ± 0.9^c^

In each column, the means with the same treatment marked with different letters are significantly different (p < 0.05, LSD test) considering the reactions of bacterial strains

### Cell surface properties

Zeta potential may be one of many determinants of cell behavior in stress conditions. Both antibiotics, NFT and FZD, cause an increase in electrokinetic potential accumulated on the surface of bacterial cells (Table 3). Only for *Pseudomonas* sp. MChB with FZD added a slight decrease, from − 16.2 to − 16.6 mV, in zeta potential was observed. In the case of treatment with pure antibiotics, the zeta potential changes slightly fluctuated by approx. 1–2 mV; the addition of saponins from *Sapindus mukorossi* caused a significant decrease in the zeta potential of bacterial cells. For each of the tested strains, there was a twofold decrease in the value of the zeta potential. Saponin molecules accumulated on the cell surface, embedding themselves in the outer cell membrane, may be responsible for such a large decrease. Amphiphilic saponin molecules can be incorporated into biological membranes by their hydrophobic (aglycon) part, while the glyconic part remains outside the outer membrane region, thus causing a decrease in the stability of the colloidal solution of bacterial cells, which causes a decrease in zeta potential (Lorent et al. 2014; Rojewska et al. 2020). Similar observations have been were made by Muniyan et al. (2017).

The Congo red adsorption test permitted evaluation of the cell adhesivity (Table 3). Comparing the results obtained for the cells treated with NFT, even 72.03% of the dye was adsorbed on cells of MChB strain. The lowest cell adhesivity value was obtained for the OS.4 strain with NFT—51.44%. It is worth noting that in the case of this series of tests, the cell adhesivity value does not drop below 50%, which proves that the dye is incorporated into the structure of bacterial cells membrane (Smułek et al. 2020). The addition of the extract caused a significant drop in the Congo Red adsorption, even below 2%. The highest value of Congo Red adsorption, of almost 30%, was obtained for the samples of MChB strain exposed to saponins only. As noted by Rojewska et al. (2020), the decrease in the Congo Red adsorption due to the presence of saponins is most likely related to the incorporation of biosurfactant molecules into the structure of the cell membrane. This results in a restriction of the space for the incorporation of the dye molecules (Rojewska et al. 2020).

## Conclusions

The results obtained in our study provide a broad perspective on the effects of nitrofuran derivatives and saponins from *Sapindus mukorossi* on the living cells. The aim of the experiments carried out was to check the possibility of enhancement of the effect of antibiotics through the supporting activity of natural surfactants on the bacterial membrane. As found, the synergistic effect was particularly evident in the systems containing both FZD and saponins, leading to a pronounced reduction in the metabolic activity of cells of s *Pseudomonas* sp. OS4 and *P. plecoglossicida* IsA strains. On the other hand, the strongest biocidal effect on *Pseudomonas* sp. MChB was observed in the samples with NFT and saponins. At the same time, the tested compounds were found to affect the fatty acid profile in the cell membranes in different ways. The cell membrane of *Pseudomonas* sp. MChB strain did not show significant modifications as a result of a contact with antibiotics and saponins, but in the cell membranes of *Pseudomonas* OS4 strain after exposition to antibiotics and surfactants were found to show dominant presence of branched fatty acids. The proportion of branched-chain fatty acids also increased significantly in the *P. plecoglossicida* IsA skeleton. The changes were evident at the level of cell surface properties. The saponins very strongly reduced the hydrophobicity of the cell surface and decreased its zeta potential. This result may indicate strong adsorption of saponins on the cell surface. In view of potential use of saponins as an antibiotic adjuvants in pharmaceutical preparations, the toxicity of antibiotics and/or saponins to colon epithelial cells was also investigated, which permitted determination of a safe dose of saponins from *S. mukorossi* at which they are not toxic and do not increase the toxicity of antibiotics. The results obtained represent significant scientific novelty and provide a basis for further research into the use of saponins for supporting the effects of antibiotics.

## Supplementary Information

Below is the link to the electronic supplementary material.

Supplementary file1 (DOCX 2041 KB)

## Data Availability

Data will be made available on request.
