# Supplementary material for: Co-interaction of nitrofuran antibiotics and the saponin-rich extract on gram-negative bacteria and colon epithelial cells
Source: World J Microbiol Biotechnol. 2023 Jun 5;39(8):221. doi: 10.1007/s11274-023-03669-2 (PMC10241687; doi:10.1007/s11274-023-03669-2)
Supplement: Supplementary file 1 — Supplementary file1 (DOCX 2041 KB) [file 11274_2023_3669_MOESM1_ESM.docx]

Supplementary Information

**Co-interaction of nitrofuran antibiotics and the saponin-rich extract on Gram-negative bacteria and colon epithelial cells**

Submitted to

*World Journal of Microbiology and Biotechnology*

by

Adam Grzywaczyk^1^, Wojciech Smułek^1^, Anna Olejnik^2^, Urszula Guzik^3^, Agnieszka Nowak^3^, Ewa Kaczorek^1^*

*^1^ Institute of Chemical Technology and Engineering, Poznan University of Technology, Berdychowo 4, 60-695 Poznan, Poland; e-mails: adam.grzywaczyk@doctorate.put.poznan.pl; wojciech.smulek@put.poznan.pl; ewa.kaczorek@put.poznan.pl*

*^2^ Department of Biotechnology and Food Microbiology, Poznań University of Life Sciences, Wojska Polskiego 48, 60-627 Poznań, Poland; e-mail: anna.olejnik@up.poznan.pl*

*^3^ Institute of Biology, Biotechnology and Environmental Protection, Faculty of Natural Science, University of Silesia in Katowice, Jagiellonska 28, 40-032 Katowice, Poland; e-mails: urszula.guzik@us.edu.pl; agnieszka.a.nowak@us.edu.pl*

*Corresponding author:

e-mail: ewa.kaczorek@put.poznan.pl, tel. +48 (61) 6652601

**E-Supplementary data**

**Growth curves**

Each bacteria strain was cultured with the presence of antibiotic in the concentration range of 0.01, 0.02, 0.05, 0.1, 0.2, 0.5, 1.0, 2.0, 5.0, 10, 20, 25, 50, 75, 100, 125, 150, 175 and 200 µg mL^-1^ of nitrofurantoin or furazolidone respectively. To 75 µg mL^-1^ of antibiotics for *P. plecoglossicida* IsA and 200 µg mL^-1^ for *Pseudomonas* sp. OS4 and MChB, *Sapindus mukorossi* extract in athe range of 30, 20, 10 and 5 µg mL^-1^ was added. Selected curves are presented.


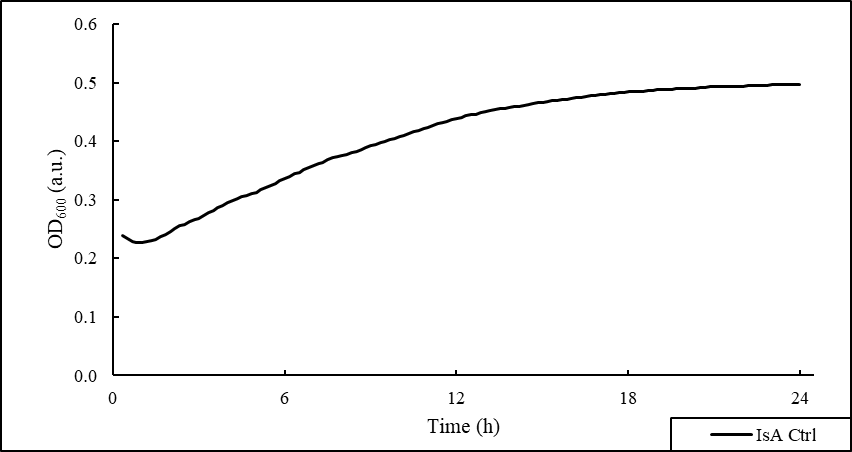


S1. Growth curve of *Pseudomonas plecoglossicida* IsA


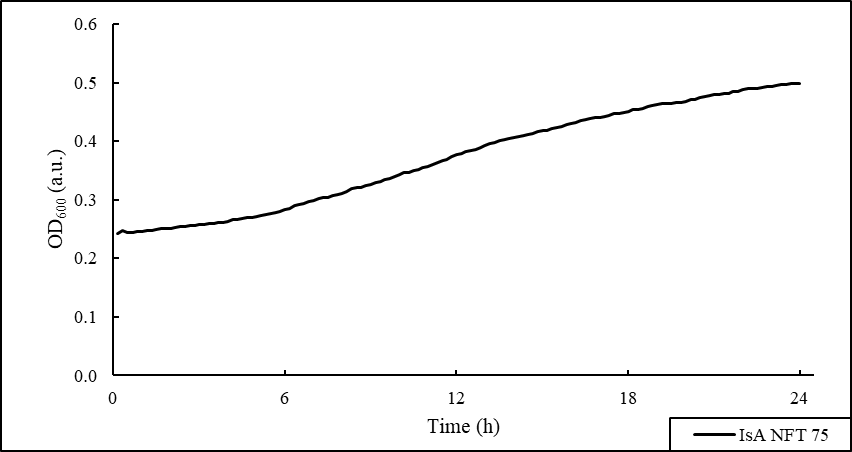


S2. Growth curve of *Pseudomonas plecoglossicida* IsA exposed to 75 µg mL^-1^ of nitrofurantoin


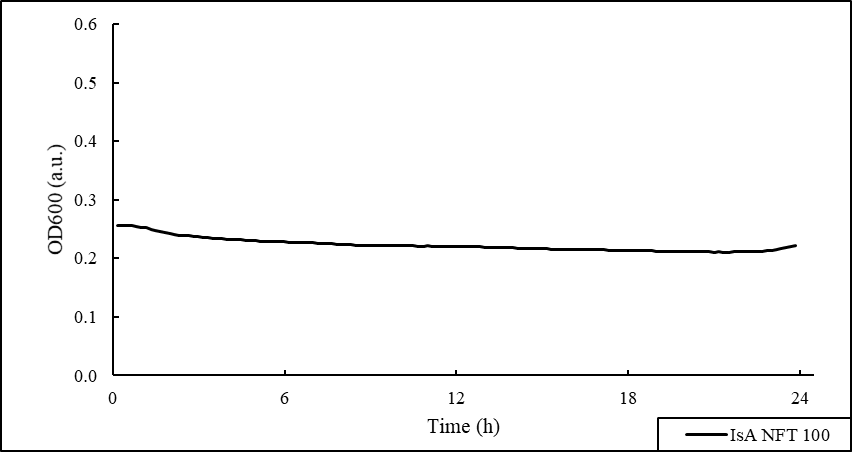


S3. Growth curve of *Pseudomonas plecoglossicida* IsA exposed to 100 µg mL^-1^ of nitrofurantoin


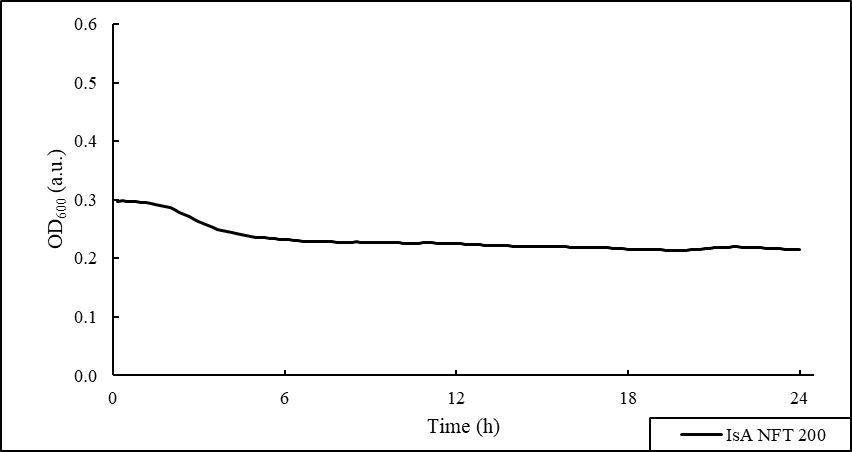


S4. Growth curve of *Pseudomonas plecoglossicida* IsA exposed to 200 µg mL^-1^ of nitrofurantoin


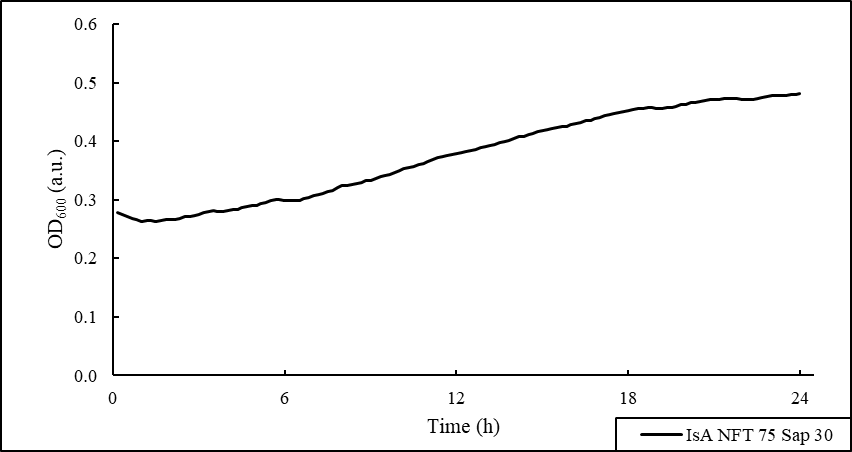


S5. Growth curve of *Pseudomonas plecoglossicida* IsA exposed to 75 µg mL^-1^ of nitrofurantoin and 30 µg mL^-1^ of *Sapindus mukorossi*


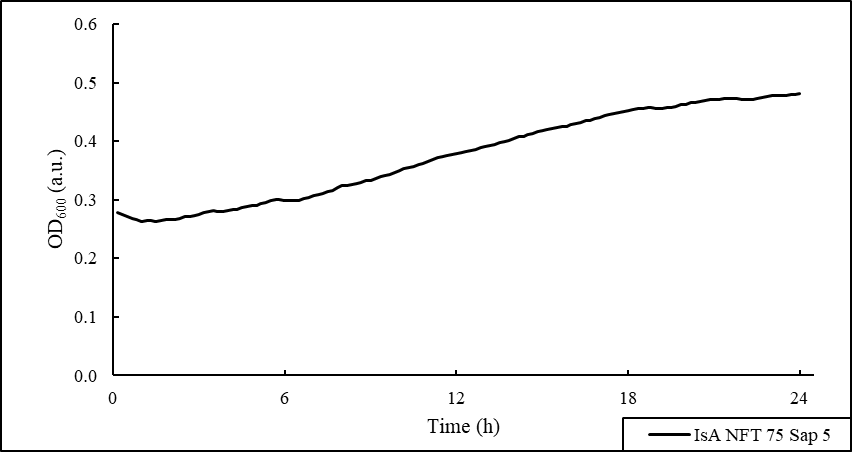


S6. Growth curve of *Pseudomonas plecoglossicida* IsA exposed to 75 µg mL^-1^ of nitrofurantoin and 5 µg mL^-1^ of *Sapindus mukorossi*


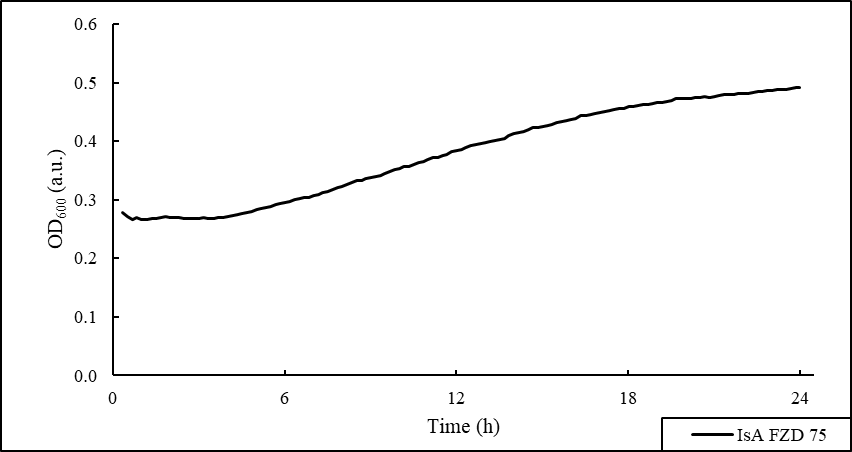


S7. Growth curve of *Pseudomonas plecoglossicida* IsA exposed to 75 µg mL^-1^ of furazolidone


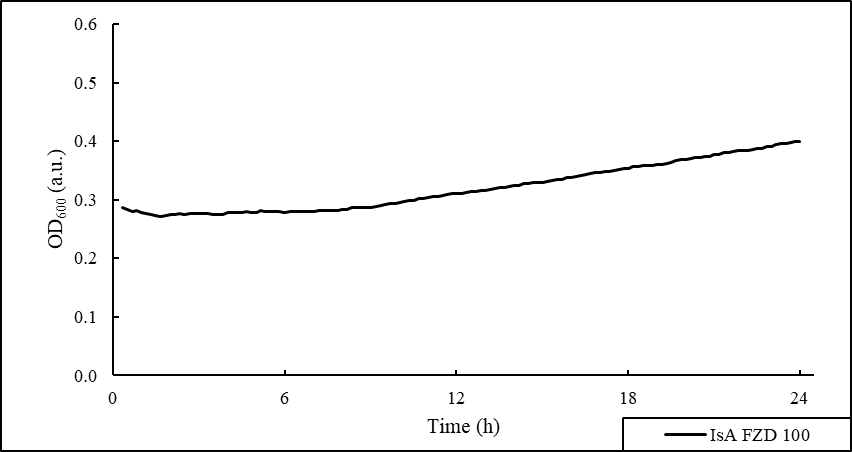


S8. Growth curve of *Pseudomonas plecoglossicida* IsA exposed to 100 µg mL^-1^ of furazolidone


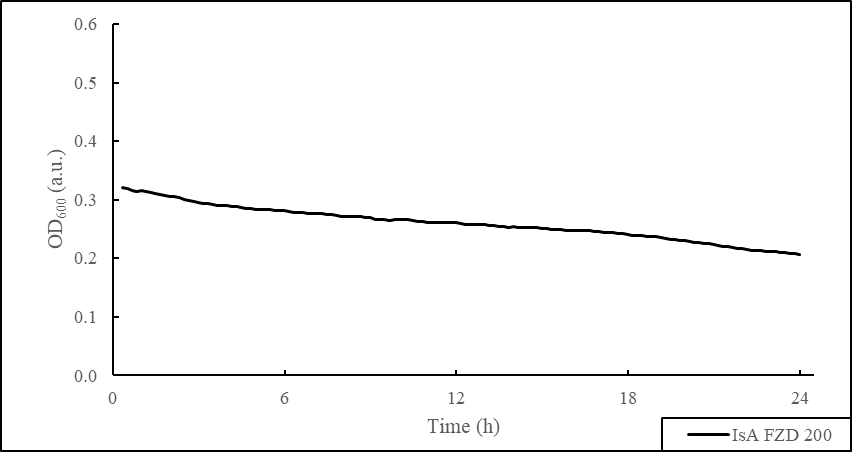


S9. Growth curve of *Pseudomonas plecoglossicida* IsA exposed to 200 µg mL^-1^ of furazolidone


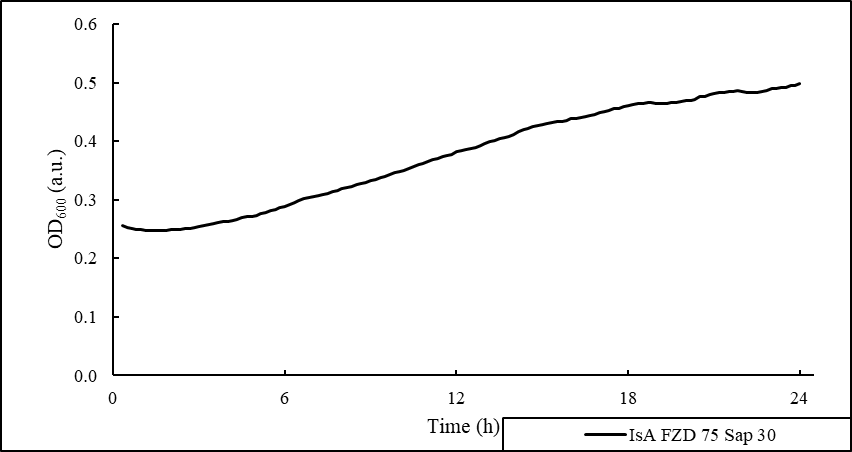


S10. Growth curve of *Pseudomonas plecoglossicida* IsA exposed to 75 µg mL^-1^ of furazolidone and 30 µg mL^-1^ of *Sapindus mukorossi*


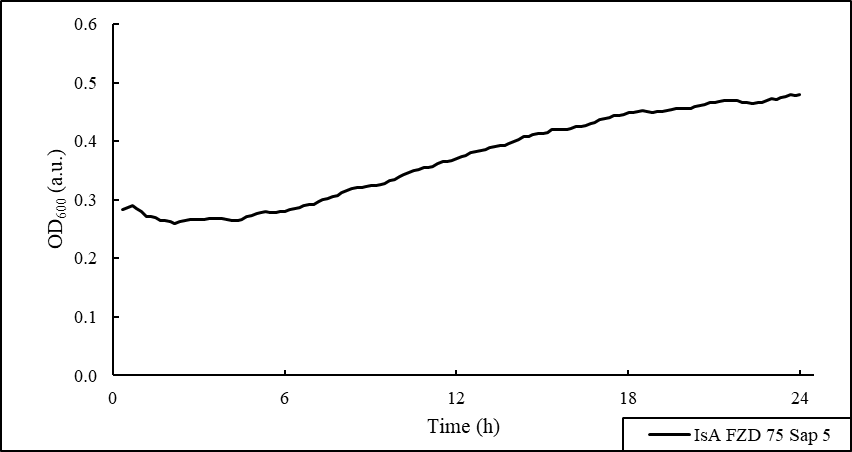


S11. Growth curve of *Pseudomonas plecoglossicida* IsA exposed to 75 µg mL^-1^ of furazolidone and 5 µg mL^-1^ of *Sapindus mukorossi*


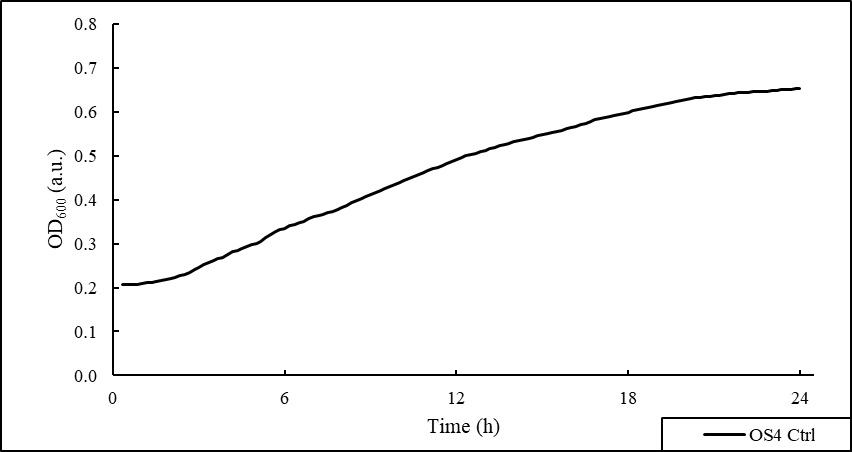


S12. Growth curve of *Pseudomonas sp.* OS4


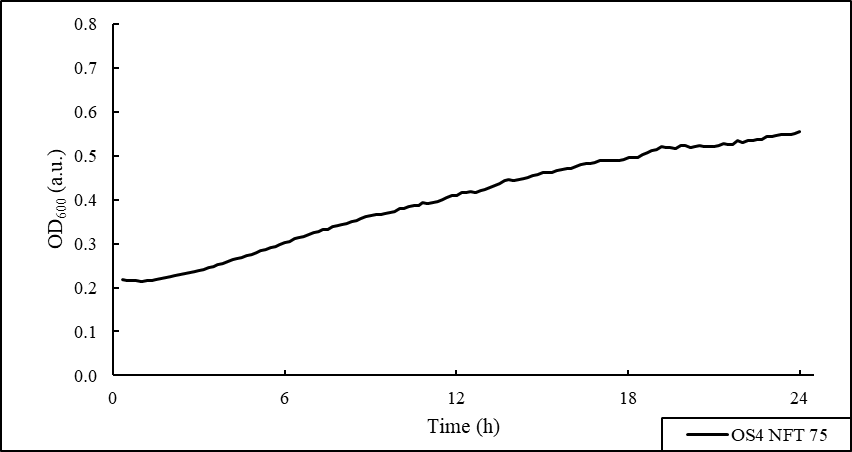


S13. Growth curve of *Pseudomonas sp.* OS4 exposed to 75 µg mL^-1^ of nitrofurantoin


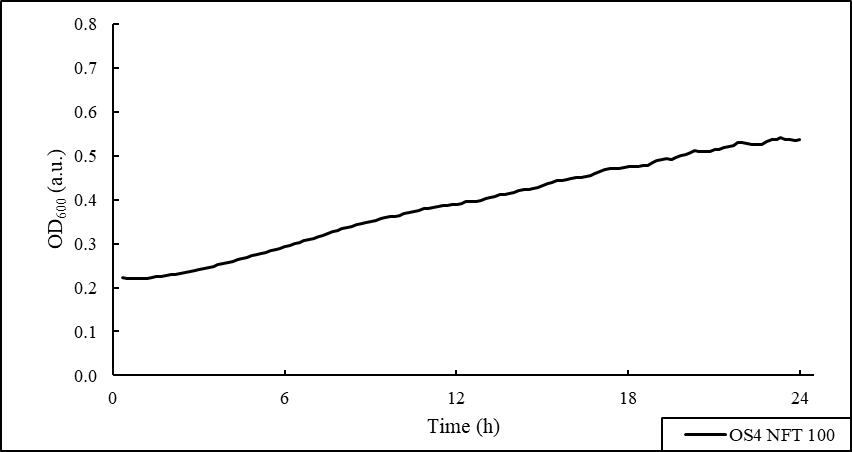


S14. Growth curve of *Pseudomonas sp.* OS4 exposed to 100 µg mL^-1^ of nitrofurantoin


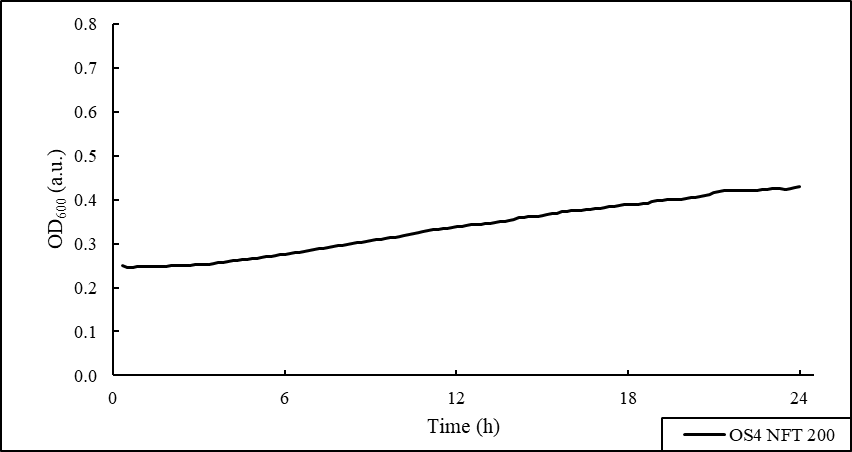


S15. Growth curve of *Pseudomonas sp.* OS4 exposed to 200 µg mL^-1^ of nitrofurantoin


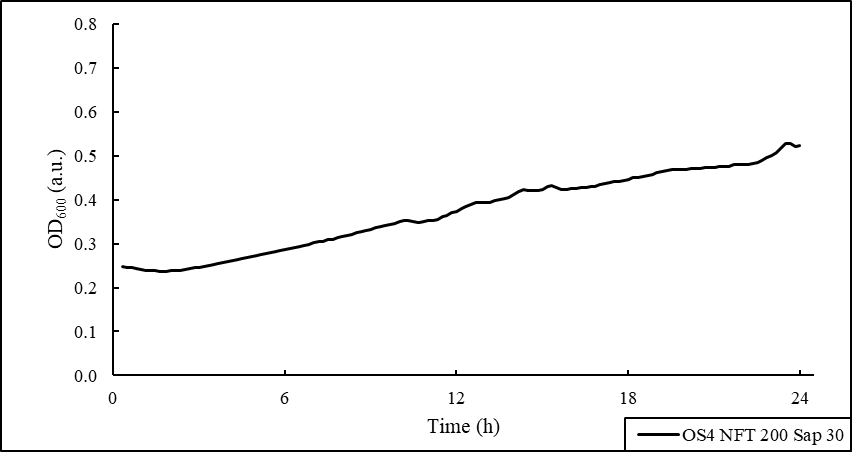


S16. Growth curve of *Pseudomonas sp.* OS4 exposed to 200 µg mL^-1^ of nitrofurantoin and 30 µg mL^-1^ of *Sapindus mukorossi*


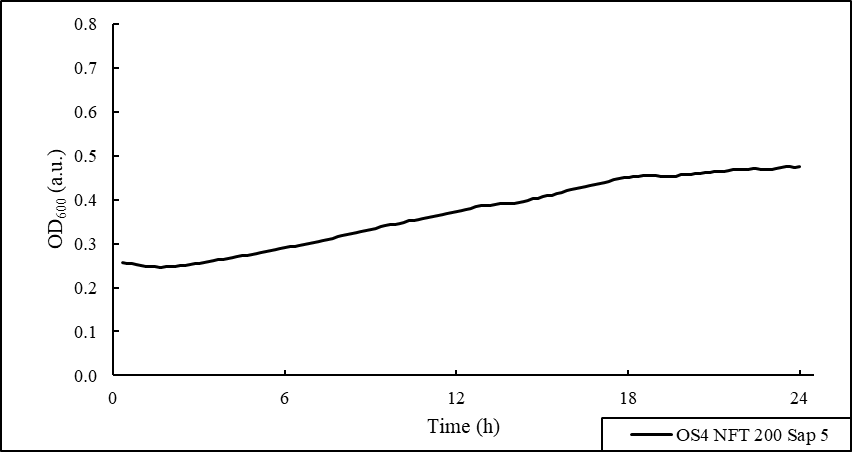


S16. Growth curve of *Pseudomonas sp.* OS4 exposed to 200 µg mL^-1^ of nitrofurantoin and 5 µg mL^-1^ of *Sapindus mukorossi*


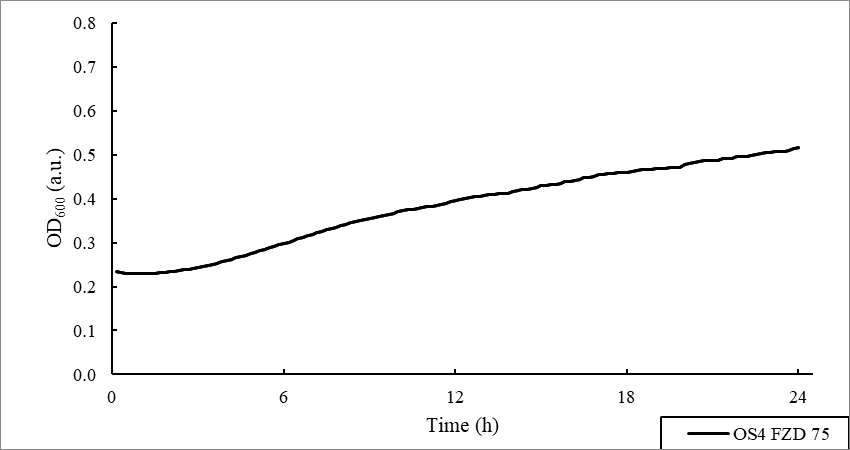


S17. Growth curve of *Pseudomonas sp.* OS4 exposed to 75 µg mL^-1^ of furazolidone


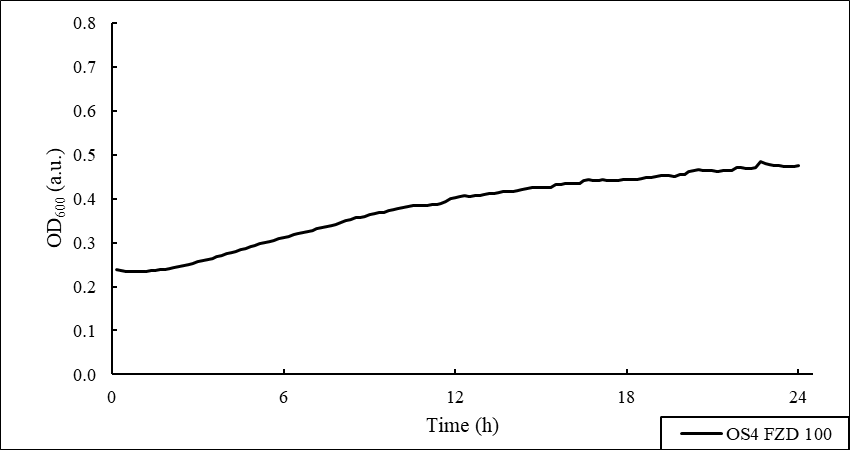


S18. Growth curve of *Pseudomonas sp.* OS4 exposed to 100 µg mL^-1^ of furazolidone


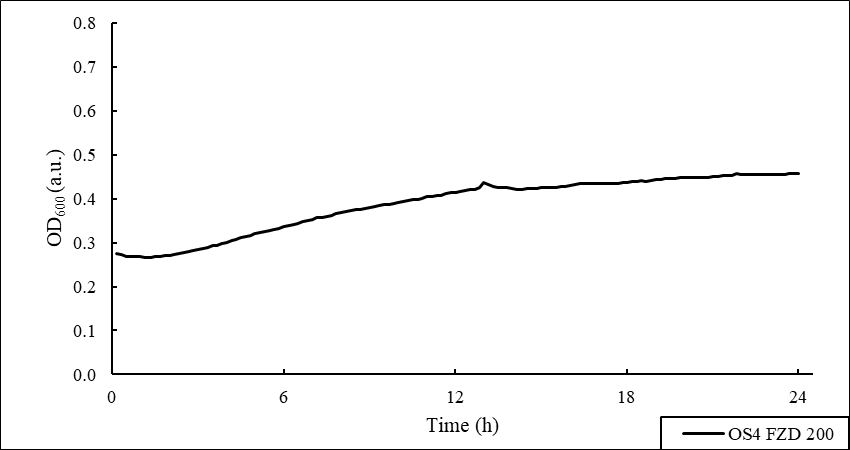


S19. Growth curve of *Pseudomonas sp.* OS4 exposed to 200 µg mL^-1^ of furazolidone


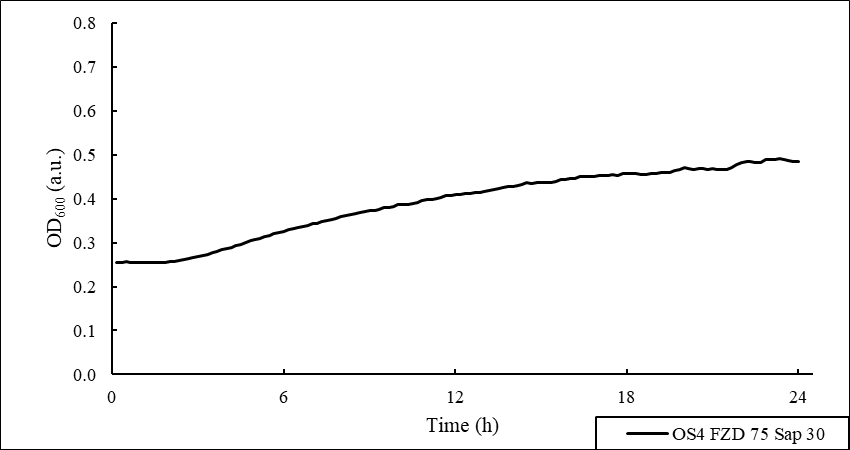


S20. Growth curve of *Pseudomonas sp.* OS4 exposed to 200 µg mL^-1^ of furazolidone and 30 µg mL^-1^ of *Sapindus mukorossi*


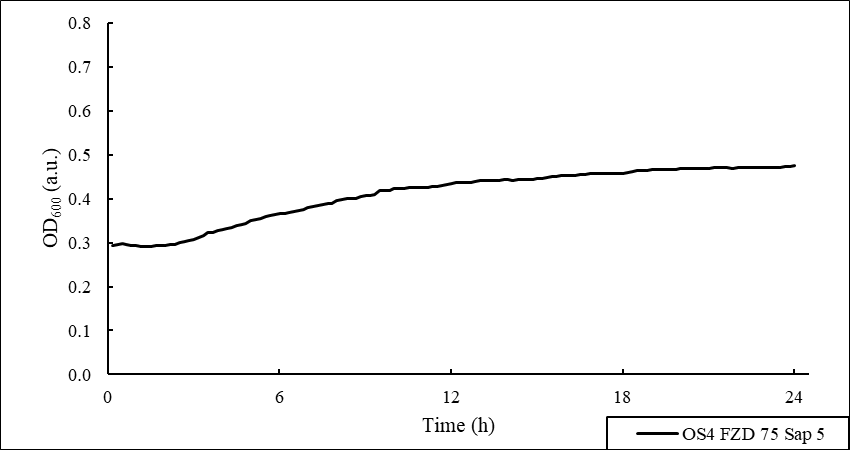


S21. Growth curve of *Pseudomonas sp.* OS4 exposed to 200 µg mL^-1^ of furazolidone and 5 µg mL^-1^ of *Sapindus mukorossi*


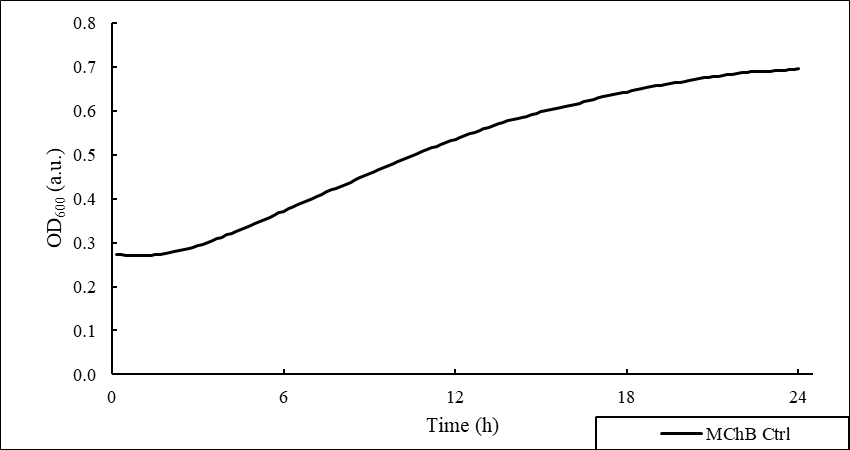


S22. Growth curve of *Pseudomonas sp.* MChB


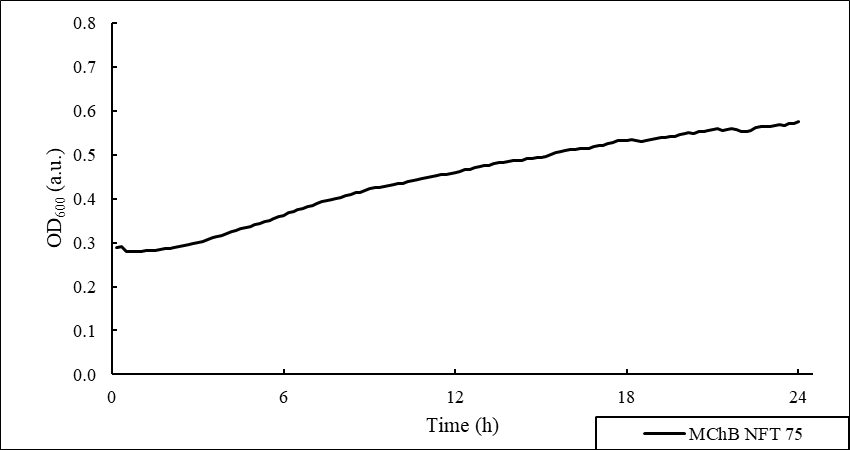


S23. Growth curve of *Pseudomonas sp.* MChB exposed to 75 µg mL^-1^ of nitrofurantoin


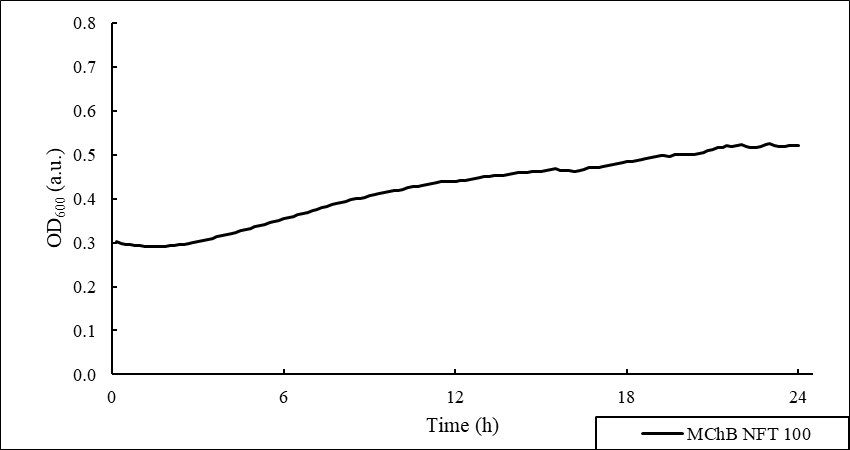


S24. Growth curve of *Pseudomonas sp.* MChB exposed to 100 µg mL^-1^ of nitrofurantoin


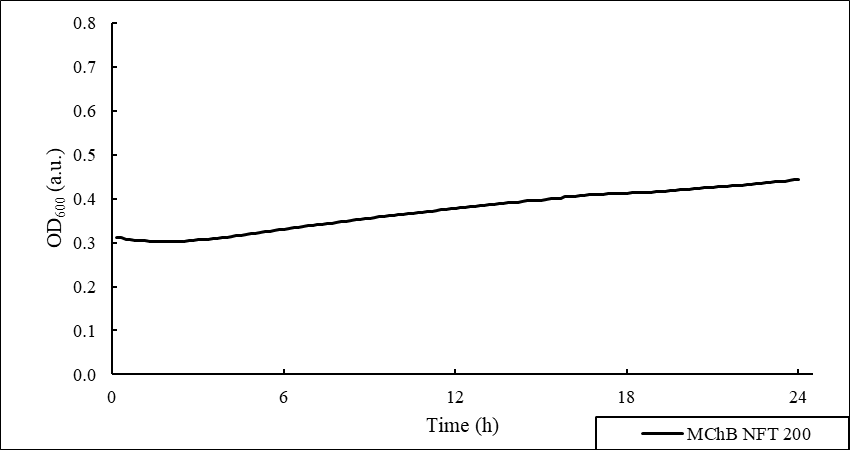


S25. Growth curve of *Pseudomonas sp.* MChB exposed to 200 µg mL^-1^ of nitrofurantoin


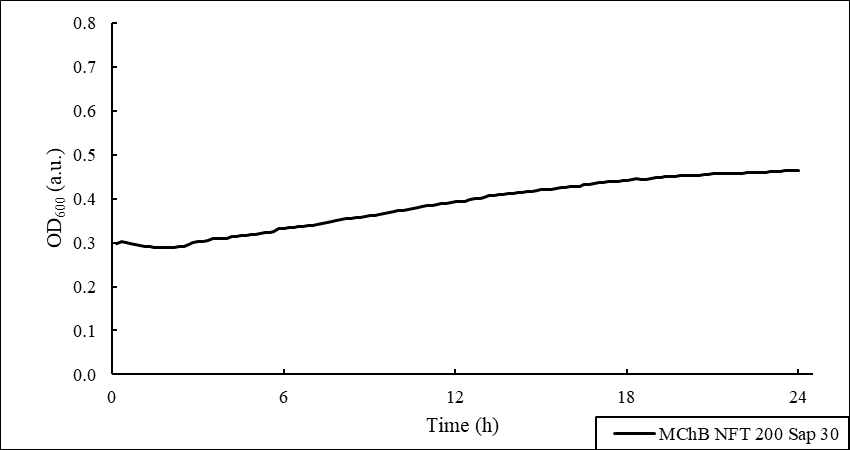


S26. Growth curve of *Pseudomonas sp.* MChB exposed to 200 µg mL^-1^ of nitrofurantoin and 30 µg mL^-1^ of *Sapindus mukorossi*


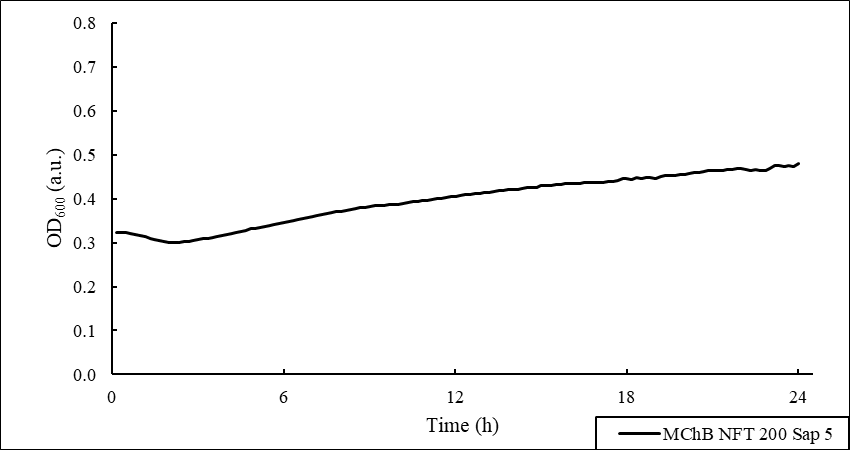


S27. Growth curve of *Pseudomonas sp.* MChB exposed to 200 µg mL^-1^ of nitrofurantoin and 5 µg mL^-1^ of *Sapindus mukorossi*


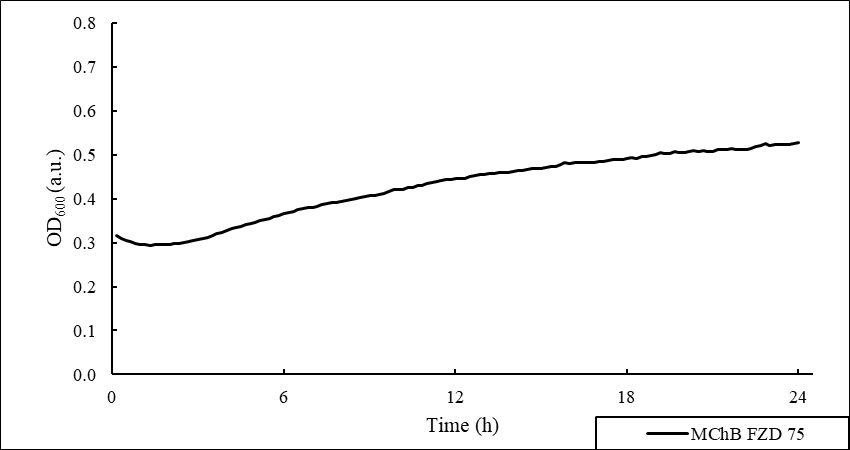


S28. Growth curve of *Pseudomonas sp.* MChB exposed to 75 µg mL^-1^ of furazolidone


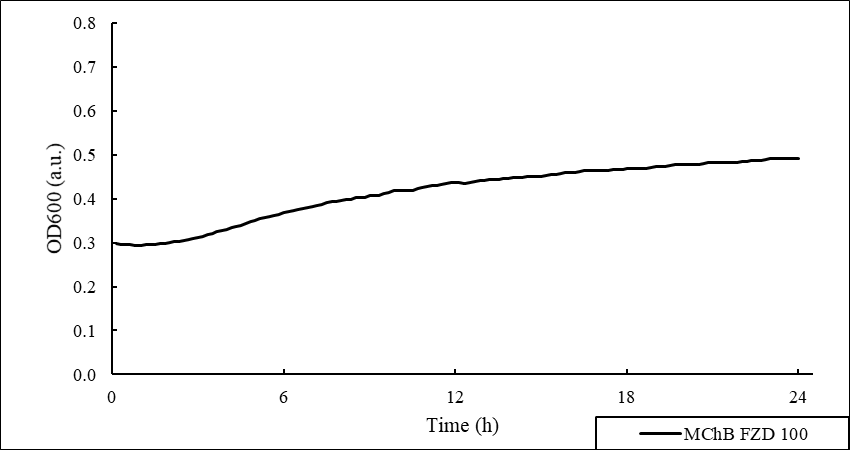


S29. Growth curve of *Pseudomonas sp.* MChB exposed to 100 µg mL^-1^ of furazolidone


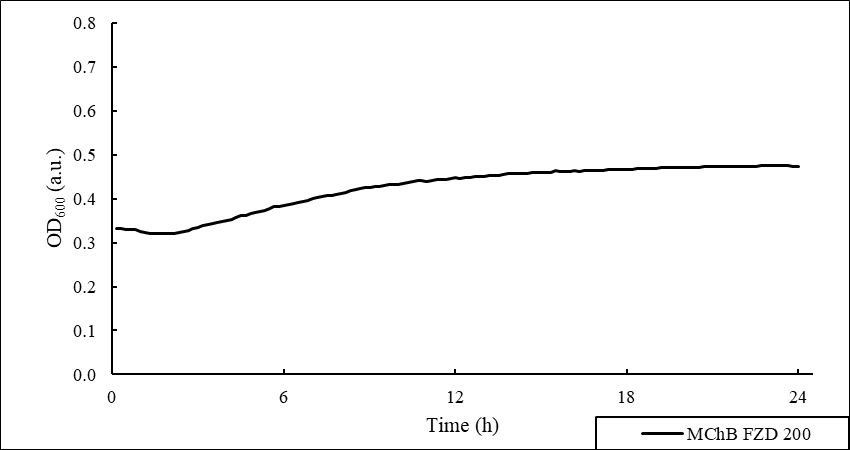


S32. Growth curve of *Pseudomonas sp.* MChB exposed to 200 µg mL^-1^ of furazolidone


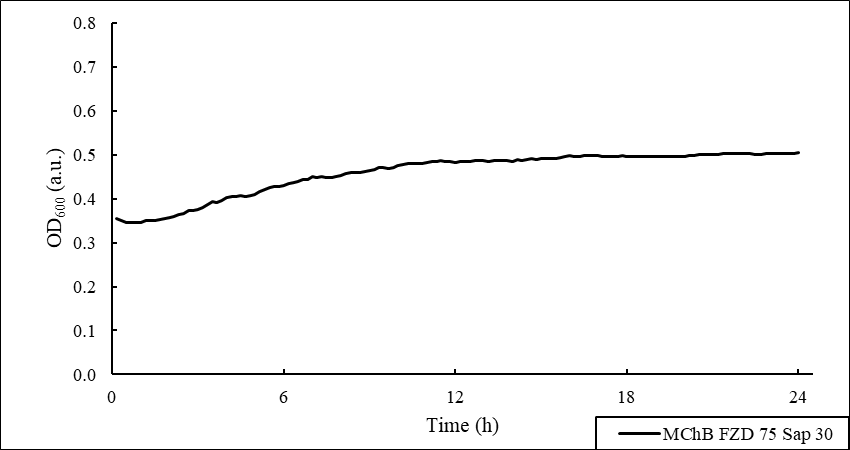


S33. Growth curve of *Pseudomonas sp.* MChB exposed to 200 µg mL^-1^ of furazolidone and 30 µg mL^-1^ of *Sapindus mukorossi*

*
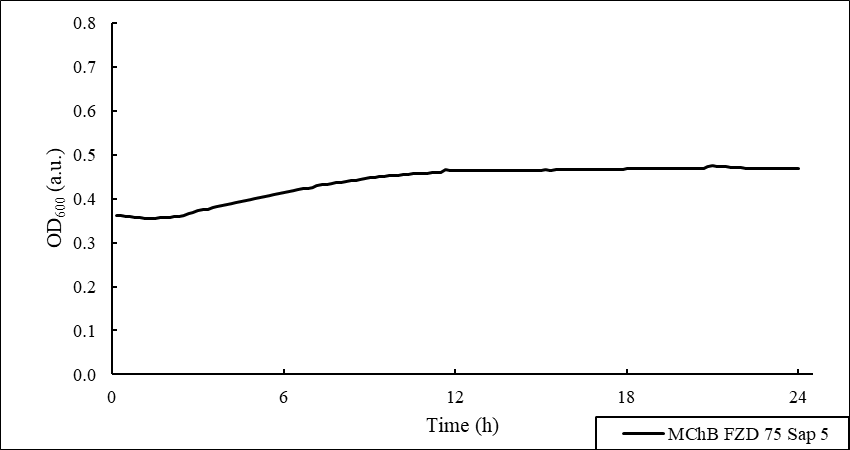
*

S34. Growth curve of *Pseudomonas sp.* MChB exposed to 200 µg mL^-1^ of furazolidone and 5 µg mL^-1^ of *Sapindus mukorossi*

**Cell topography**


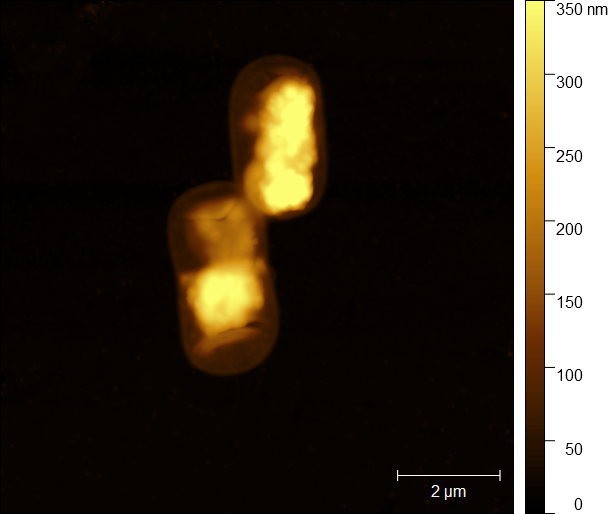


S35. Surface two-dimensional height images illustrating the *Pseudomonas plecoglossicida* IsA topography, Control sample


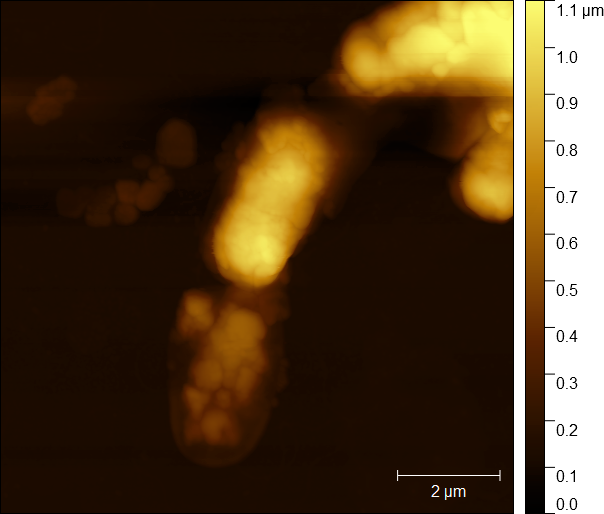


S36. Surface two-dimensional height images illustrating the *Pseudomonas plecoglossicida* IsA topography, Exposed to 5 µg mL^-1^ of NFT


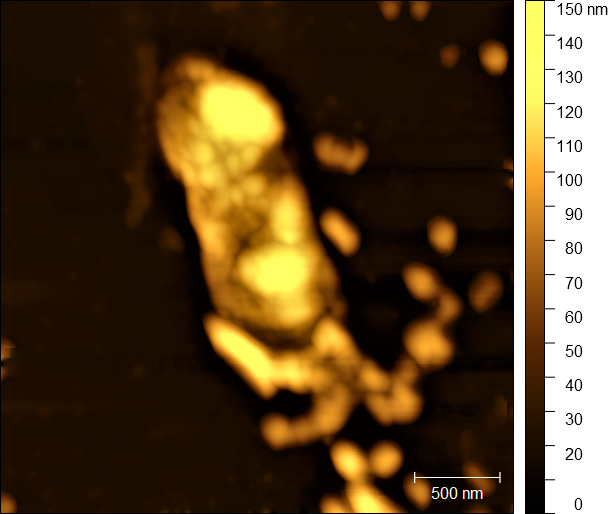


S37. Surface two-dimensional height images illustrating the *Pseudomonas plecoglossicida* IsA topography, Exposed to 5 µg mL^-1^ of NFT and 10 µg mL^-1^ of saponins


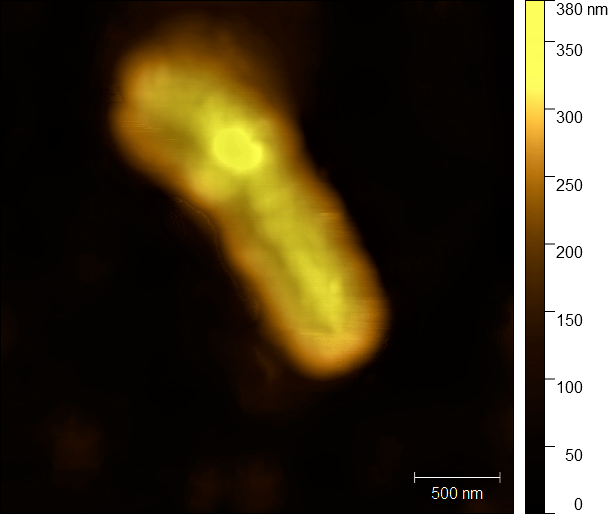


S38. Surface two-dimensional height images illustrating the *Pseudomonas sp.* OS4 topography, Control sample


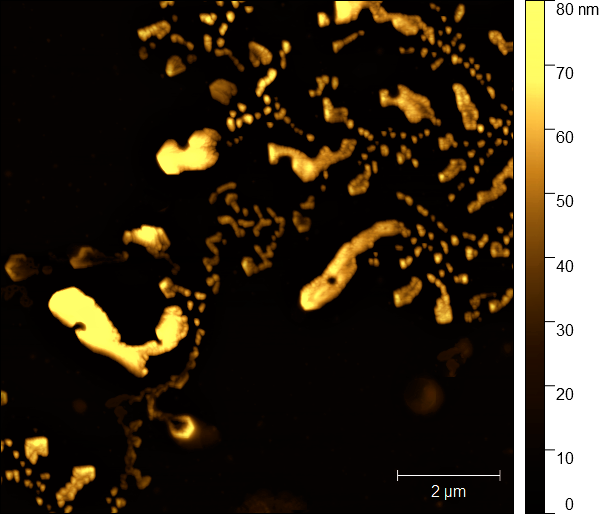


S39. Surface two-dimensional height images illustrating the *Pseudomonas sp.* OS4 topography, Exposed to 5 µg mL^-1^ of NFT


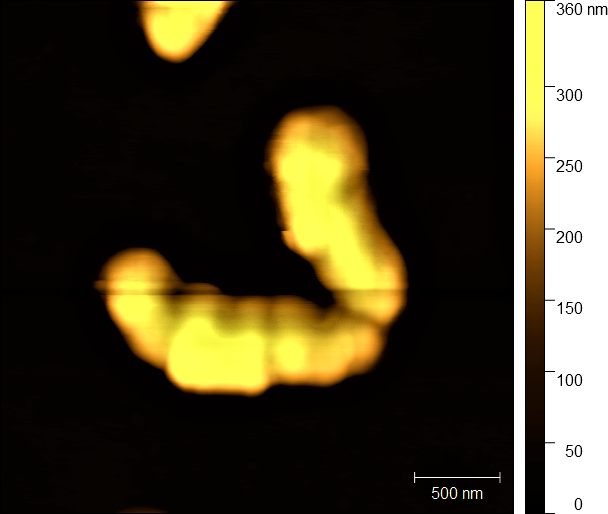


S40. Surface two-dimensional height images illustrating the *Pseudomonas sp.* OS4 topography, Exposed to 5 µg mL^-1^ of NFT and 10 µg mL^-1^ of saponins


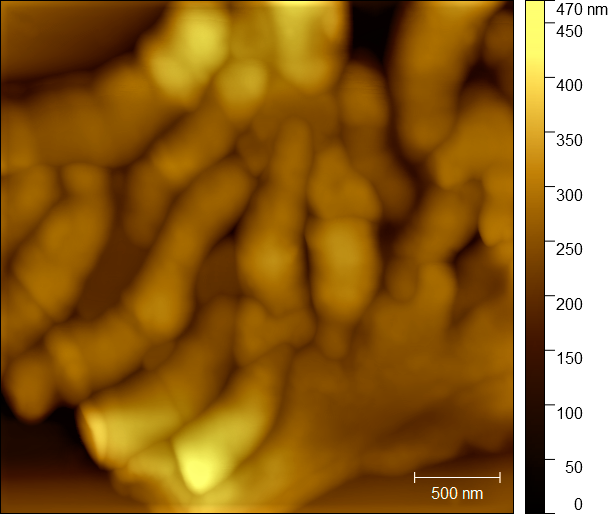


S41. Surface two-dimensional height images illustrating the *Pseudomonas sp.* MChB topography, Control sample


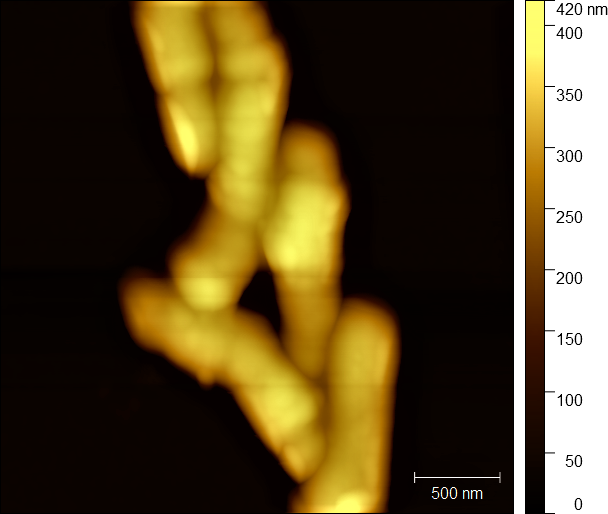


S42. Surface two-dimensional height images illustrating the *Pseudomonas sp.* MChB topography, Exposed to 5 µg mL^-1^ of NFT


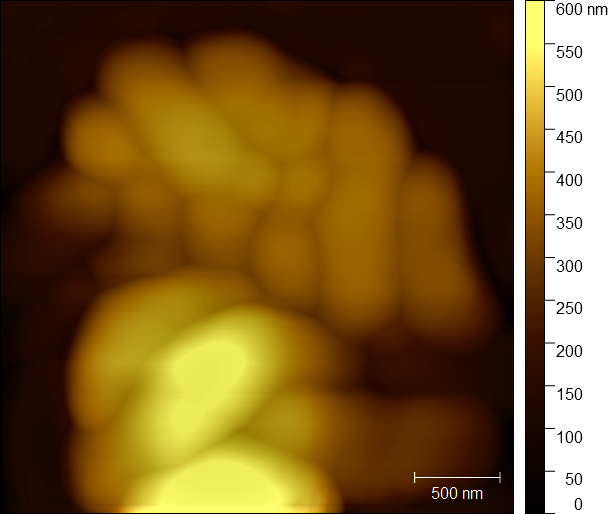


S43. Surface two-dimensional height images illustrating the *Pseudomonas sp.* MChB topography, Exposed to 5 µg mL^-1^ of NFT and 10 µg mL^-1^ of saponins
